# High Keratin-7 Expression in Benign Peri-Tumoral Prostatic Glands Is Predictive of Bone Metastasis Onset and Prostate Cancer-Specific Mortality

**DOI:** 10.3390/cancers14071623

**Published:** 2022-03-23

**Authors:** Charles Dariane, Sylvie Clairefond, Benjamin Péant, Laudine Communal, Zhe Thian, Véronique Ouellet, Dominique Trudel, Nazim Benzerdjeb, Feryel Azzi, Arnaud Méjean, Marc-Olivier Timsit, Manon Baurès, Jacques-Emmanuel Guidotti, Vincent Goffin, Pierre I. Karakiewicz, Anne-Marie Mes-Masson, Fred Saad

**Affiliations:** 1 Centre de Recherche du Centre Hospitalier de l’Université de Montréal (CRCHUM), Institut du Cancer de Montréal (ICM), Montréal, QC H2X 0A9, Canada; sylvie.clairefond@usask.ca (S.C.); benjamin.peant.chum@ssss.gouv.qc.ca (B.P.); laudine.communal@gmail.com (L.C.); veronique.ouellet.chum@ssss.gouv.qc.ca (V.O.); dominique.trudel.chum@ssss.gouv.qc.ca (D.T.); feryel.azzi.chum@ssss.gouv.qc.ca (F.A.); anne-marie.mes-masson@umontreal.ca (A.-M.M.-M.); fred.saad@umontreal.ca (F.S.); 2 Department of Urology, Hôpital Européen Georges-Pompidou, Université de Paris, F-75006 Paris, France; arnaud.mejean@aphp.fr (A.M.); marc-olivier.timsit@aphp.fr (M.-O.T.); 3 Institut Necker Enfants Malades (INEM), Inserm U1151, Université de Paris, F-75006 Paris, France; manon.baures@inserm.fr (M.B.); jacques-emmanuel.guidotti@inserm.fr (J.-E.G.); vincent.goffin@inserm.fr (V.G.); 4 Cancer Prognostics and Health Outcomes Unit, Division of Urology, University of Montréal Health Center, Montréal, QC H2X 0A9, Canada; zhe.tian@mail.mcgill.ca (Z.T.); pierre.karakiewicz@umontreal.ca (P.I.K.); 5 Department of Pathology, Centre Hospitalier Universitaire Lyon Sud, F-69310 Pierre-Bénite, France; nazim.benzerdjeb@chu-lyon.fr; 6 Department of Pathology and Cellular Biology, Centre Hospitalier de l’Université de Montréal (CHUM), 1000 rue St Denis, Montréal, QC H2X 0C1, Canada; 7 Department of Surgery, Université de Montréal, Montréal, QC H3C 3J7, Canada; 8 Department of Medicine, Université de Montréal, Montréal, QC H3C 3J7, Canada

**Keywords:** prostate cancer, prognostic biomarker, survival, metastasis-free survival, immunofluorescence, benign peri-tumoral prostatic glands, keratin-7

## Abstract

**Simple Summary:**

Keratin-7 overexpression has been associated with poor prognosis in several cancers. To determine its prognostic relevance in prostate cancer, cohorts of patients presenting with localized or advanced tumors were investigated by either immunohistochemistry or immunofluorescence. Keratin-7 immunostaining was absent in localized tumors and rare in advanced tumors. By contrast, it was abundantly detected in basal cells within benign glands surrounding localized tumors. Importantly, high keratin-7 expression in benign peri-tumoral glands was correlated with shorter bone metastasis-free survival and increased risk of cancer-specific mortality. This study establishes keratin-7 expression in localized prostate cancer specimens as a promising biomarker of disease progression.

**Abstract:**

Background: New predictive biomarkers are needed to accurately predict metastasis-free survival (MFS) and cancer-specific survival (CSS) in localized prostate cancer (PC). Keratin-7 (KRT7) overexpression has been associated with poor prognosis in several cancers and is described as a novel prostate progenitor marker in the mouse prostate. Methods: KRT7 expression was evaluated in prostatic cell lines and in human tissue by immunohistochemistry (IHC, on advanced PC, *n* = 91) and immunofluorescence (IF, on localized PC, *n* = 285). The KRT7 mean fluorescence intensity (MFI) was quantified in different compartments by digital analysis and correlated to clinical endpoints in the localized PC cohort. Results: KRT7 is expressed in prostatic cell lines and found in the basal and supra-basal compartment from healthy prostatic glands and benign peri-tumoral glands from localized PC. The KRT7 staining is lost in luminal cells from localized tumors and found as an aberrant sporadic staining (2.2%) in advanced PC. In the localized PC cohort, high KRT7 MFI above the 80th percentile in the basal compartment was significantly and independently correlated with MFS and CSS, and with hypertrophic basal cell phenotype. Conclusion: High KRT7 expression in benign glands is an independent biomarker of MFS and CSS, and its expression is lost in tumoral cells. These results require further validation on larger cohorts.

## 1. Introduction

Prostate cancer (PC) is the malignant tumor with the highest incidence in men and ranks second to lung cancer in terms of mortality in the United States [1]. PC lethality is a corollary of distant metastasis, due to the eventual development of castration-resistant disease under androgen deprivation therapy (ADT) [2]. While the vast majority of PCs are organ-confined at diagnosis [3], a significant proportion of these tumors relapse following surgery or radiation therapy [4,5]. This necessitates salvage therapy to prevent or limit the development of distant metastases [4]. For example, up to 20% of men with intermediate-grade PC will experience biochemical recurrence (BCR) within 3 years of definitive local therapy [6], which portends an aggressive clinical course. This implies the presence of occult pelvic or metastatic cells at the time of surgery, which are not accurately predicted using current risk-stratification guidelines based on standard clinical prognostic factors (i.e., Gleason grade group, pre-treatment serum concentration of prostate-specific antigen, and clinical T category) [7].

Other prognostic models, including histological data, have been developed to predict BCR or metastasis-free survival (MFS), such as the Kattan nomogram [8], the cancer of the prostate risk assessment (CAPRA) score [9], and the STAR-CAP prognostic system [10]. However, the majority of these studies focus on weak surrogates of cancer-specific survival (CSS) (e.g., BCR which is an early and easily measured endpoint) [11] and a recent meta-analysis validated the MFS as a more meaningful clinical endpoint and the only identified surrogate endpoint for overall survival (OS) in localized PC [12]. New predictive biomarkers are then needed to complement available clinical, histological, and radiological data (i.e., magnetic resonance imaging) [13] and to accurately predict MFS and CSS.

Keratins (KRTs) are useful biological markers for determining the origin and differentiation status of specific epithelial cells in normal tissues as well as their malignant counterparts [14]. Neoplasms largely retain the keratin profile during malignant transformation, but the aberrant expression of KRTs such as keratin-7 (KRT7) can also indicate a poor prognosis for patients [15]. KRT7 is a type II cytokeratin located in intermediate filaments. It is mainly expressed in glandular epithelia where it exhibits a cytoplasmic pattern. KRT7 is detected in many tissues and its overexpression in tumors was associated with disease progression in several cancer types [15,16,17,18]. There is no similar data currently available for KRT7 expression in PC. KRT7 expression was not described in the normal prostatic epithelium and its expression in PC was reported to be an exceptional finding [19]. One study suggested increased KRT7 reactivity in high-grade tumors [20]. In the mouse prostate, KRT7 was recently described as a novel prostate progenitor marker, and authors have suggested that label-retaining stem cells co-expressing candidate stem cell markers (such as SCA-1, TROP-2, CD133, CD44, c-KIT, and KRT7) could function as cancer-initiating and relapse-driver cells in murine PC [21]. In humans, the identification of cells with stem/progenitor properties and their ability to resist castration are of major interest as such cells may be involved in resistance to ADT in advanced PC [22]. KRT7 has been identified as a putative stemness factor related to cancer stem cells in other cancer such as liver cancer [23]. Together, these data stress the need to assess the relevance of KRT7 expression in the human prostate and in PC.

In this study, KRT7 expression was investigated in healthy prostate tissue from a young donor and in benign and malignant prostate tissue at different stages. KRT7 expression was absent in localized tumors and rare in advanced PC tissue from metastatic patients. By contrast, KRT7 was abundantly detected in normal and benign peri-tumoral glands, mostly in the basal cell compartment. We found that the intensity of KRT7 expression in benign peri-tumoral glands was associated with bone metastasis-free survival and PC-specific mortality.

## 2. Materials and Methods

### 2.1. Cell Lines

PC cell lines 22Rv1, DU145, LNCaP, PC3, PZ-HPV-7, and RWPE-1 were obtained from the American Type Culture Collection (ATCC, Manassas, VA, USA). Jurkat T lymphoma cells were kindly provided by Dr. Lapointe (CRCHUM).

### 2.2. Exploratory Cohorts of Patients

Antibody staining optimizations were performed using a formalin-fixed paraffin-embedded (FFPE) tissue micro-array (TMA) designed for immunolabelling optimization, gathering 18 cores from various prostatic tissues: acinar carcinoma from radical prostatectomies (*n* = 10), intra-ductal carcinoma (*n* = 1), proximal and distal adjacent benign tissue (*n* = 4), trans-urethral prostatic chips from a metastatic patient (*n* = 1), and trans-urethral chips from benign prostatic hyperplasia (*n* = 2).

To assess baseline KRT7 expression, normal healthy prostates from an autopsy TMA were obtained from the CRCHUM biobank (*n* = 51 donors). The localization of the KRT7 staining was validated on FFPE tissue slides from radical prostatectomies, from patients with ductal carcinomas and concomitant adjacent acinar pattern (*n*= 16 patients), selected from the CHUM biobank using the term “ductal” in pathology reports from patients operated for a localized PC between 2012 and 2018. To assess the KRT7 expression in advanced PC, specimens of transurethral resection of the prostate (TURPs) (*n* = 91) performed between 1989 and 2006, from metastatic patients, were identified from the CHUM prostate biobank. One to three tissue cores (malignant and/or adjacent benign) from each specimen were embedded on this separate TMA. All subjects gave informed consent for inclusion in the PC biobank of the CHUM, affiliated to the Réseau de la recherche sur le cancer (RRCancer).

### 2.3. Validation Cohort of Localized PC Patients

The TMA named TerryFox123 (TF123) is composed of primary PC tissue from 285 patients who underwent radical prostatectomy (RP) at the Centre hospitalier de l’Université de Montréal (CHUM, Montréal, QC, Canada) between 1993 and 2006, with no preoperative androgen deprivation therapy or chemotherapy and at least 5 years of post-operative follow-up. This TF123 was composed of a total of six TMA blocks. For each patient, 2 adjacent benign duplicate 0.6 mm cores and 2 tumoral duplicate cores were available, as previously published [24,25]. The composition of the cohort is described in Supplementary Table S1. All subjects gave informed consent for inclusion in the PC biobank of the CHUM, affiliated to the Réseau de la recherche sur le cancer (RRCancer), before participating in the study. The study was conducted in accordance with the Declaration of Helsinki, and the protocol was approved by the Ethics Committee de la recherche du CHUM (#2013-4072, CE 12.216-BSP). The time to BCR was defined as the interval between the date of the RP and the date of a prostate-specific antigen (PSA) level above 0.2 ng/mL and rising, or when a decision to proceed with additional therapy was made. Bone metastasis onset and death from PC defined the metastasis-free survival (MFS) and the cancer-specific survival (CSS).

### 2.4. Cell Culture

Jurkat T lymphoma cells and 22Rv1, DU145, LNCaP, PC3 were maintained in RPMI 1640 medium (Wisent Inc., St-Bruno, QC, Canada), supplemented with 10% fetal bovine serum (Gibco©, Thermo Fisher Scientific, Waltham, MA, USA), 0.454 µg/mL amphotericin B (Wisent Inc.), and 90 µg/mL gentamycin sulphate (Wisent Inc., St-Bruno, QC, Canada).

Prostatic PZ-HPV-7 and RWPE-1 cell lines were maintained in Keratinocyte-serum free medium (K-SFM) (Gibco©, Thermo Fisher Scientific, Waltham, MA, USA), supplemented, for each 500 mL of medium, with 2.5 µg of EGF human recombinant and 25 mg of Bovine pituitary extract (Gibco©, Thermo Fisher Scientific, Waltham, MA, USA), and 5 mL of Penicillin 10000 IU-Streptomycin 10000 µg/mL (450-201 EL, Wisent Inc., St-Bruno, QC, Canada). A Defined Trypsin Inhibitor (Gibco©, Thermo Fisher Scientific, Waltham, MA, USA) was specifically used after Trypsin use since the K-SFM medium is trypsin-inhibitor free.

### 2.5. Paraffin Processing and Embedding of Cell Line Pellets

PC cell line pellets were used as a control on the TMA and prepared as previously described [26]. This method was developed to fix and embed cell suspensions in paraffin using HistoGel™ (Thermo Fisher Scientific, Waltham, MA, USA) to ensure a high cell density per core when arrayed on a TMA. The embedded cell suspensions in paraffin allowed to normalize the conditions in which the patient tissues FFPE samples were tested.

### 2.6. Western Blots Analysis

Confluent cells were harvested and incubated with lysis buffer (1% Triton, 10% glycerol, 50 mM Tris, 2 mM EDTA, and 150 mM NaCl) supplemented with fresh protease and phosphatase inhibitors (PIA32961, Thermo Fisher Scientific, Waltham, MA, USA) for 30 min on ice. After centrifugation, protein concentrations of whole-cell extracts were determined by Bradford assays (Genesys 10S UV-Vis Spectrophotometer, Thermo Fisher Scientific, Waltham, MA, USA). Proteins (30 µg) were separated using 4–15% Mini-PROTEAN^®^ TGX™ Precast gels (Bio-Rad, Mississauga, ON, Canada) and transferred onto nitrocellulose membranes using the Trans-Blot^®^ Turbo™ Transfer System (Bio-Rad, Mississauga, ON, Canada). Membranes were immunoblotted with primary antibodies, either mouse monoclonal anti-KRT7 (1:250, Clone OV-TL 12/30, NBP1-22539, Novus Biologicals, Toronto, ON, Canada) or rabbit monoclonal anti-KRT7 (1:250, Clone SP52, ab183344, Abcam Inc., Cambridge, UK) antibodies. Each primary antibody was diluted in Tris-buffered saline tween 20 (TBS-T) supplemented with 5% fat-free skim milk powder (SKI400.1, Bioshop Canada Inc., Burlington, ON, Canada). Either β-actin (AC-15, ab6276, Abcam Inc., Cambridge, UK) or GAPDH (0411, sc-44724, Santa Cruz Biotechnology, Dallas, TX, USA) were used as loading control for immunoblots involving mouse or rabbit anti-KRT7 antibodies, respectively. Protein expression was detected with HRP-conjugated secondary antibodies (EMD Millipore Corp., Burlington, MA, USA) and immunoreactive bands were revealed by enhanced chemiluminescence (ECL™ Prime Western Blotting Detection Reagents, RPM2232, Amersham™, Cytiva, UK).

### 2.7. Immunohistochemistry (IHC) on FFPE Tissues

After standard deparaffinization, tissue sections (4 µm) were stained with the Benchmark XT automated stainer (Ventana Medical Systems, Roche, Rotkreuz, Switzerland). Antigen retrieval was obtained using Cell Conditioning #1 solution (Ventana Medical System Inc., Tris-EDTA buffer, pH 7.8) for 60 min at 95 °C. Pre-diluted mouse monoclonal anti-KRT7 antibody (1:200, OV-TL 12/30, DAKO Omnis, Agilent, Mississauga, ON, Canada) and rabbit monoclonal anti-KRT7 antibody (1:150, Clone SP52, ab183344, Abcam Inc., Cambridge, UK) were manually added to the slides and incubated at 37 °C (32 min for OV-TL 12/30 or 60 min for SP52 antibodies). Reactions were visualized using either the DAB Detection Kit for OV-TL 12/30 antibody or the Alkaline Phosphatase (AP) Detection Kit for SP52 antibody (Ventana Medical System Inc., Oro Valley, AZ, USA). Counterstaining was achieved with hematoxylin and bluing reagent (Ventana Medical System Inc., Oro Valley, AZ, USA). All sections were scanned with an Aperio Verso 200 slide scanner microscope using a 20 × 0.8 NA objective and resolution of 0.275 MPP (Leica Biosystems, Buffalo Grove, IL, USA), allowing images to be viewed at a 40× magnification. The Image Scope software (Leica Biosystems, Buffalo Grove, IL, USA) was used for image visualization.

### 2.8. Immunofluorescence (IF) on FFPE Tissues

TMA sections (4 µm) were subjected to a semi-automatic IF multiplex staining protocol using the Benchmark XT automated stainer after standard deparaffinization. The antigen retrieval was performed in Cell Conditioning #1 solution.

Primary KRT7 antibodies, either mouse monoclonal anti-KRT7 antibody (1:250, Clone OV-TL 12/30, NBP1-22539, Novus Biologicals, Toronto, ON, Canada), or mouse monoclonal auto-conjugated anti-KRT7 antibody (1:150, Clone OV-TL 12/30 [Alexa Fluor^®^750], NBP2-47940 AF750, Novus Biologicals, Toronto, ON, Canada), were diluted in phosphate-buffered saline (PBS 10X, Multicell, Wisent, Inc., St-Bruno, QC, Canada), added manually to slides and incubated at 37 °C for 60 min. To properly identify basal cells and distinguish them from luminal cells, a cocktail containing monoclonal antibodies against mouse p63 (1:2, DAK-p63, IR662, DAKO, Agilent, Santa Clara, CA, USA) and mouse anti-cytokeratin high molecular weight (CKHMW) (1:50, 34bE12, CLSG36689-05, Cedarlane, Fremont, CA, USA) was applied for 60 min to the section, concomitantly with the primary auto-conjugated anti-KRT7 antibody incubation.

The following manual steps were performed away from light after removing the slides from the automated stainer. Slides were blocked with a protein block serum-free universal solution (DAKO, Agilent, Mississauga, ON, Canada) for 20 min. The mouse monoclonal anti-KRT7 antibody OV-TL 12/30, NBP1-22539 was detected with a Cy5™ goat anti-mouse IgG (1:250, Thermo Fisher Scientific, Waltham, MA, USA) diluted in PBS containing 1% bovine serum albumin (BSA, A3294, Sigma-Aldrich, St Louis, MO, USA). The secondary antibody step was omitted when using the auto-conjugated antibody OV-TL 12/30 [Alexa Fluor^®^750], NBP2-47940 AF750. For detection of p63 and CKHMW, a secondary fluorescent antibody Alexa Fluor™ 546 donkey anti-mouse IgG (1:250, Thermo Fisher Scientific, Waltham, MA, USA) diluted in PBS/BSA 1% was added for 45 min.

To avoid cross-reactivity, slides were blocked overnight with 250 µL of PBS containing 50 µL of a mouse-on-mouse (MOM) Ig blocking reagent (1:5 dilution, VECT MKB-2213, MJS BioLynx Vector Laboratories Inc., Burlingame, CA, USA). To detect the epithelium, a highly sensitive cocktail of antibodies against cytokeratins 8 (1:100, TS1, MA5-14228, Thermo Fisher Scientific) and 18 (1:100, DC-10, sc-6259, Santa Cruz Biotechnology, Dallas, TX, USA) was used, diluted in PBS, and incubated on day 2 at room temperature for 60 min after overnight MOM. This step was followed by incubation with a secondary fluorescent antibody Alexa Fluor™ 488 goat anti-mouse IgG (1:250, Thermo Fisher Scientific, Waltham, MA, USA) for 45 min.

Following DAPI staining to identify nuclei, each slide was incubated for 15 min at room temperature with a 0.1% solution of Sudan Black B (Research Organics, Cleveland, OH, USA) in 70% ethanol to quench tissue autofluorescence. Between each step, all slides were washed twice with 1 X PBS. Finally, slides were mounted with coverslips using Fluoromount™ Aqueous Mounting Medium (F4680, Millipore-Sigma, Oakville, ON, Canada). Slides were stored overnight at room temperature and scanned the following day. A negative control slide was processed in parallel and incubated with PBS instead of the primary antibodies, then processed with the appropriate secondary antibodies, including an Alexa Fluor™ 750 goat anti-mouse IgG (1:250, Thermo Fisher Scientific, Waltham, MA, USA) for the auto-conjugated KRT7 control. All slides were scanned within 24 h with an Olympus Optical microscope BX61VSF (Olympus, Shujuku, Tokyo, Japan) using a 20 × 0.75 NA objective and a resolution of 0.325 µm (Olympus Canada Inc., Richmond Hill, ON, Canada), linked to the Olympus OlyVIA^®^ 2.9 image viewer software (Build 13771, Olympus, xvViewer.exe).

### 2.9. IF Staining Digital Image Analyses and Pre-Processing of Scoring Data

Fluorescent staining of the different markers was quantified by automated digital image analyses (VisiomorphDP software, Visiopharm, Hoersholm, Denmark), as previously described [27]. VisiomorphDP semi-automated algorithms first used DAPI staining to enable the delimitation of the “whole tissue”, secondly KRT8/18 staining to discriminate the “epithelium” and “stroma” Regions Of Interest (ROI) in each TMA core, and thirdly p63/CKHMW to discriminate “basal compartment” from “luminal compartment” inside the epithelium. Marker expression was quantified in each image pixel of each ROI to calculate the mean fluorescence intensity (MFI) within the ROI.

Data pre-processing was performed with quality control of the tissue cores as described previously [24,25]. To accurately compare all of the TMAs (6 separate blocks), an MFI normalization was performed to compensate for the observed differences related to slide scanning and imaging in the average fluorescence intensity between each slide arising from the 6 blocks. Normalization to the mean MFI for KRT7 and in each compartment was achieved with the following equation example: [Mean of MFI KRT7 in epithelium (all 6 slides of the series)] / [Mean of MFI KRT7 in epithelium (slide of interest)] = Normalizing core ratio. This normalizing ratio was applied to KRT7 in the corresponding compartment: KRT7 MFI in basal compartment (core of interest) × normalizing core ratio = KRT7 MFI normalized in basal compartment.

### 2.10. Statistical Analysis of MFI Values from the TMA TF123

After normalization, the mean of the duplicate core MFI values for each patient (2 benign cores and 2 tumoral cores) was calculated prior to subsequent statistical analysis using the SPSS Statistics 26.0 software package (SPSS Inc., Chicago, IL, USA). To compare KRT7 expression between benign and tumoral cores and between tissue compartments (epithelium versus stroma, luminal versus basal epithelial cells), a Mann-Whitney test was used on GraphPad Prism software V6 (GraphPad, La Jolla, CA, USA).

The data were first analyzed using the median and mean fluorescence intensity. Then they were analyzed as quartiles (<25th percentile, between 25–50th, between 50–75th or >75th percentile), and then quintiles (<the 20th percentile, between 20–40th, between 40–60th, between 60–80th or > 80th percentile) to explore data trends and identify the threshold percentile providing the most significant dichotomization into groups of low and high expression (either above the 20th, 40th, 60th or 80th percentile). Survival curves plotted were established using the Kaplan–Meier method coupled with a log-rank test, as well as Cox regression analysis tested for statistical significance in observed differences. KRT7 expression was systematically analyzed by its continuous and dichotomized expression values. Univariate and multivariate (Cox regression) analyses were used to estimate the hazard ratio (HR) for KRT7 expression. Bivariate correlations were analyzed using the Pearson correlation coefficient. A two-sided *p*-value < 0.05 was considered statistically significant.

## 3. Results

### 3.1. Validation of Immunostaining Procedures

Antibody specificity was validated and the baseline expression of KRT7 in prostatic cell lines with different species of antibodies (mouse OV-TL 12/30 and rabbit SP52) was determined by Western blot assay on whole-cell extracts (Supplementary Figure S1A). Both mouse and rabbit antibodies showed specific and similar bands around the expected 51 kDa molecular weight (Supplementary Figure S2), as previously described [18]. It was noted that the KRT7 protein level was higher in castration-resistant PC cell lines (DU145, PC3) and non-malignant prostate cell lines (PZ-HPV-7 and RWPE-1) when compared to hormone-sensitive PC cell lines (22Rv1 and LNCaP). Jurkat T lymphoma cells were used as negative controls with no KRT7 expression since hematological cell lines do not express cytokeratin markers.

Since these antibodies were also used in FFPE human tissues in this study, the antibody specificity was validated on cell line derivatives by IF assay. Cell pellets from these cell lines were fixed and paraffin-embedded on a TMA to perform KRT7 immunostaining using mouse OV-TL 12/30 antibody (Supplementary Figure S1B). The detection level of KRT7 in cell pellets from the above-mentioned cell lines was generally similar to that observed in the cognate Western Blot, with a negative staining for the hormone-sensitive PC and Jurkat cell lines and other cell lines presenting a positive staining (Supplementary Figure S1B). The antibodies were therefore considered specific.

### 3.2. Characterization of KRT7 Expression in Different Tissue Samples from Exploratory Cohorts

#### 3.2.1. Optimization TMA (*n* = 18)

**Figure 1:**
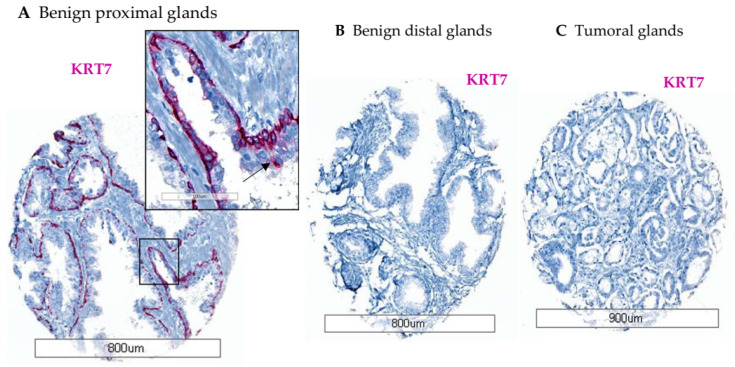
Examples of KRT7 staining by IHC using KRT7 rabbit SP52 clone on FFPE prostatic tissues from optimization-TMA. (**A**) Positive KRT7 staining was found in basal and supra-basal (arrow) cells from benign proximal peri-tumoral glands, (**B**) and negative staining in either benign distal glands, (**C**) or in a tumor core from acinar carcinoma. Alkaline phosphatase (AP) enzyme was used as a label for KRT7 detection in IHC.

To optimize KRT7 staining, an FFPE TMA containing 18 cores from various prostatic tissues was used. By IHC, the KRT7 expression was only detected in the basal cell compartment from all tumor-adjacent benign glands with rabbit monoclonal SP52-KRT7 antibody (Figure 1A). The KRT7 staining was cytoplasmic, mostly localized in basal cells and supra-basal cells. The staining in benign glands was intense in cores from the proximal peri-tumoral zone and was almost negative in distal benign glands (Figure 1B). In tumoral cores composed of luminal cells, no KRT7 expression was found (Figure 1C), similarly to the description by some authors of prostate carcinoma as usually KRT7-negative [22]. Overall, KRT7 staining was not detected in tumoral glands in localized PC, which is consistent with loss of basal cells and expansion of luminal cells during tumorigenesis [28], although no basal marker was added to KRT7 staining in IHC (compared to IF).

#### 3.2.2. Tissue Slides from Healthy Prostate (*n* = 51)

To assess the potential relevance of KRT7 expression in basal cells as a PC prognostic marker, the baseline expression of KRT7 in normal tissue (from an autopsy TMA, *n* = 51 healthy men with 24 cases showing tissue of sufficient quality for reliable analysis) was evaluated with a multi-staining IF assay including auto-conjugated OV-TL 12/30 KRT7 antibody (Cy7 pink) (Supplementary Figure S3A,B). Again, the KRT7 expression was found to be restricted to basal cells (p63/CKHMW-positive cells) from normal glands, with a moderate intensity (KRT7 staining found in 20/24 cores); but no luminal expression (KRT8/18-positive cells) was detected. Some glands with a basal hypertrophy pattern showed a more intense KRT7 staining, mimicking urothelial metaplasia (Supplementary Figure S3B). This led to the assumption that KRT7 moderate expression in basal cells was physiological since it was present in normal healthy autoptic prostate tissue with no underlying known disease.

#### 3.2.3. Whole Tissue Slides from Localized PC (*n* = 16)

**Figure 2:**
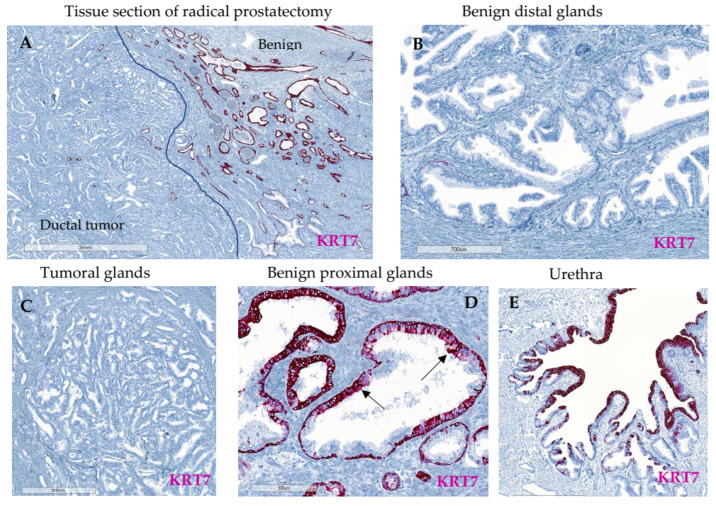
Examples of KRT7 staining by immunohistochemistry (IHC) using KRT7 rabbit SP52 clone on FFPE prostatic tissues section of radical prostatectomy from a patient (Patient #1) with prostatic acinar and ductal carcinoma. (**A**,**D**) The KRT7 staining was found augmented in peri-tumoral benign glands proximal to the tumor (blue line defining the separation between tumor and benign peri-tumoral glands), (**A**,**C**) but negative inside the tumor, (**B**) and decreased in benign glands located distally. (**D**) After magnification, positive KRT7 staining was confirmed mainly in the basal compartment from benign proximal peri-tumoral glands, with a supra-basal staining identified with arrows, (**C**) and negative in tumoral ductal glands. (**E**) Positive staining was found in cells of the urethral urothelium, as a control.

Since KRT7 staining has been proposed to be associated with high-risk tumors [20], FFPE tissue slides from radical prostatectomies of 16 patients with highly aggressive features (i.e., ductal carcinomas) were used and stained with rabbit monoclonal SP52-KRT7 antibody. This allowed the localization of KRT7 to be characterized and the visualization of the specific staining spatial distribution. Interestingly, tumoral glands from either acinar or ductal sections were negative for KRT7 (Figure 2A,C). Similar to the 18 yo-patient TMA, KRT7 expression was found in peri-tumoral benign glands in the basal or supra-basal compartment (Figure 2A,D). Observation of KRT7 staining was augmented in benign proximal peri-tumoral glands adjacent to the tumor compared to distal benign glands, obtained from a tissue block where no cancer was detected or transitional zone (Figure 2B). As a control, positive KRT7 staining was observed in the urethra’s urothelium (Figure 2E) as already described [29]. Two additional patients exhibiting similar KRT7 distribution between the compartments are illustrated in Supplementary Figure S4.

#### 3.2.4. Advanced PC TMA (*n* = 91)

To assess whether KRT7 expression was different in locally advanced PC from metastatic patients, the expression of KRT7 was explored by IHC (using mouse monoclonal OV-TL 12/30-KRT7 antibody) in a cohort of transurethral resection of prostate (TURPs) specimens from 91 patients organized in a TMA. Among this cohort, KRT7 was detected in only 2/91 (2.2%) patients. KRT7 was expressed in luminal tumoral undifferentiated cells from a hormone-sensitive (patient #2) and a castration-resistant PC (CRPC) patient (patient #1) with a Gleason score of 5 + 4 on both tissues (Supplementary Figure S5A). Of note, patient #2 with KRT7-positive cells had adjacent benign cores available on the same TMA, and these cores were, surprisingly, KRT7-negative in basal cells (Supplementary Figure S5B). Together, these results led us to explore the potential prognostic role of KRT7 staining either in basal adjacent benign glands and to focus on a cohort of patients with localized PC with either benign or tumoral cores available.

### 3.3. Expression of KRT7 in a Cohort of Localized PC (n = 285)

**Figure 3:**
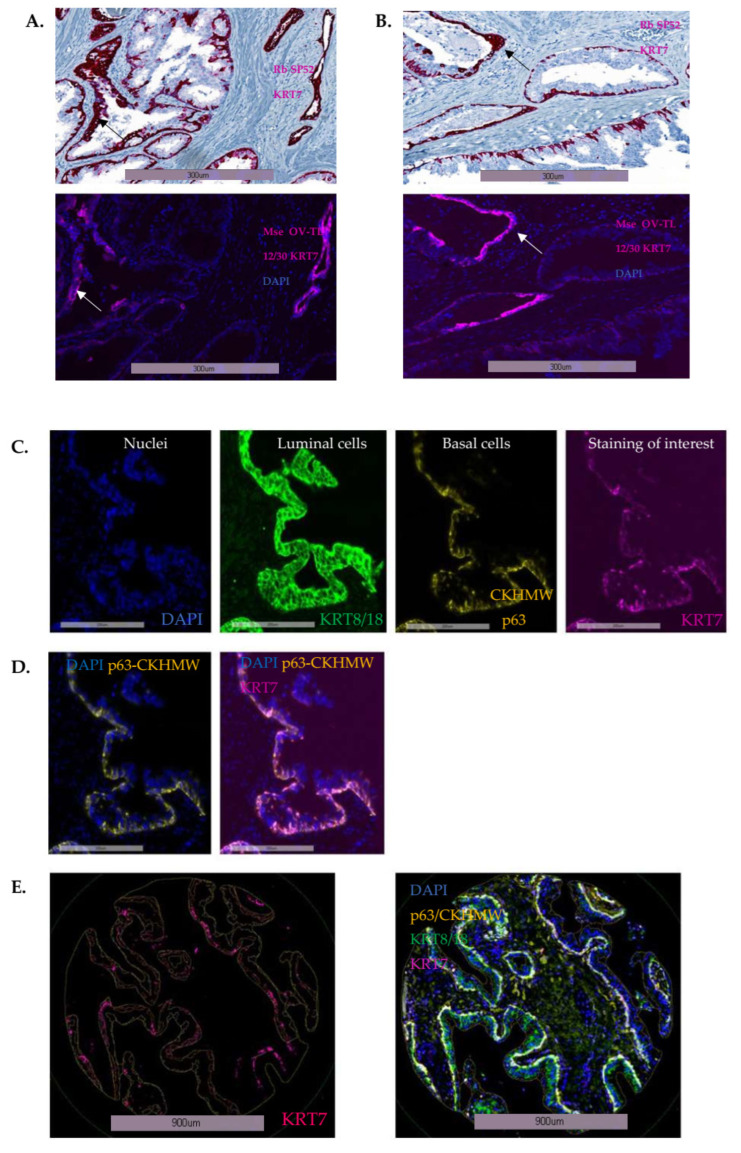
Comparison of KRT7 staining using two different antibodies and two different staining strategies (IF/IHC) on serial slides of radical prostatectomies from localized PC, and details of the digital image protocol optimized for multiplex staining and biomarker analyses. (**A**,**B**) Concordance of KRT7 staining between the rabbit SP52 staining in IHC and the mouse OV-TL 12/30 staining in IF, in two separate areas from tissue section of radical prostatectomies. Supra-basal staining is indicated with arrows. (**C**) Multiplex staining of TMA cores discriminates nuclei (DAPI, blue), luminal cells (KRT8/18, FITC-488 green), basal cells (p63/CKHMW, TRITC-546 yellow), and the studied biomarker KRT7 (mouse OV-TL 12/30 KRT7, Cy7 pink) in benign peri-tumoral glands, (**E**) with superimposed images corresponding to merged co-staining. (**D**) p63 and KRT7 positively stained in basal cells without cross-over staining.

The prognostic value of KRT7 expression was assessed in a cohort of radical prostatectomy specimens (*n* = 285 patients) from well-balanced localized PC with clinical/ pathological characteristics detailed in Supplementary Table S1 [24,25]. The incidence of biochemical recurrence (BCR) at 5 years was 33% (94 patients), and 9.8% (28 patients) of the cohort had an onset of bone metastasis leading in 6.3% of cases (18 patients) to a prostate-cancer specific death. This cohort was richly annotated, and with long-term follow-up allowed for the evaluation of ultimate PC endpoints such as MFS and CSS. First, in order to verify the specificity of the antibodies from both species, we confirmed that a similar pattern of basal cell KRT7 staining was found either in IHC using the rabbit SP52 antibody or with IF using the mouse OV-TL 12/30 antibody (Figure 3A,B). The intensity of signal, however, was lower in IF, which may be due to lower sensitivity of the IF antibody and/or to signal amplification of the secondary detection system in IHC. In agreement, KRT7 staining in cells from the supra-basal layer was more pronounced in IHC (see below, arrows). The selection of different regions of interest was performed using mask algorithms and a multi-staining approach (Figure 3C–E) (a basal cell mask with p63/CKHMW and a luminal cell mask with KRT8/18 staining cocktail), as earlier reported [24,27]. KRT7 expression was mainly detected in basal and supra-basal cells from benign epithelial glands (Figure 3C–E).

**Figure 4:**
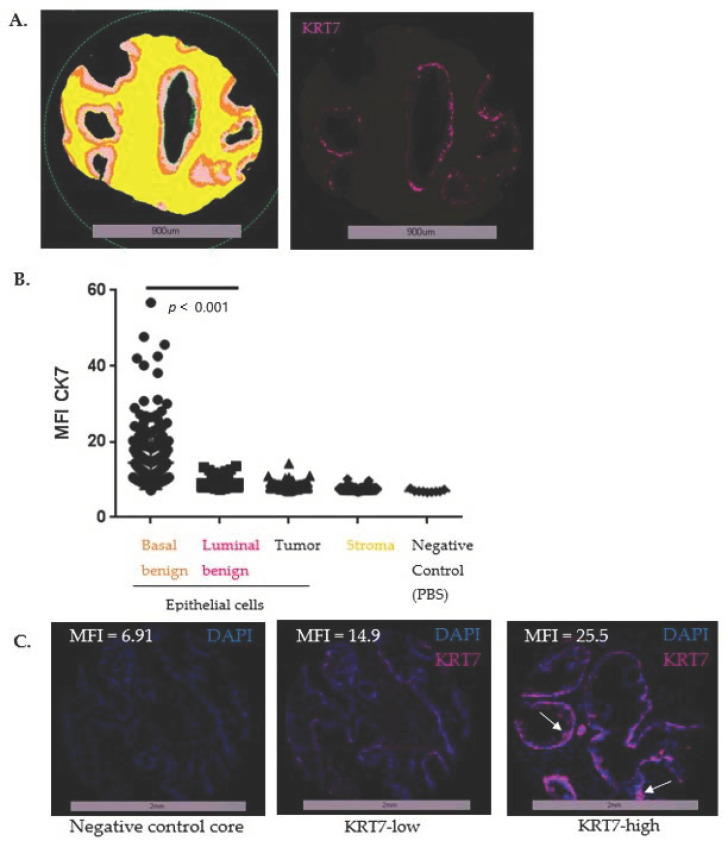
Characterization of KRT7 expression in stroma and epithelium from benign and tumoral glands. (**A**) Detection of KRT7 IF staining in basal cells from benign glands. KRT7 (detected using KRT7 OV-TL 12/30) was mainly expressed in the basal compartment of benign glands. (**B**) Quantification of KRT7 expression in each tissue compartment. Mean fluorescent intensities (MFI) were quantified by VisiomorphDP software and normalized using the ratio of the whole core. For each patient, expression levels were calculated using the average mean of two cores. (**C**) Examples of KRT7-low and KRT7-high staining in epithelial cells of benign glands adjacent to the tumor. Negative control core corresponded to the quantification of MFI in cores when IF was performed with only secondary antibodies (Cy7 antibody anti-mouse). KRT7-low corresponded to cores with intensity under the 80th percentile in basal benign cells (MFI < 17.56) and KRT7-high was established at an intensity higher than the 80th percentile (MFI > 17.56) in basal benign cells, after quintiles evaluation of MFI. Supra-basal staining is indicated with arrows. Magnification 20×.

Using an already published algorithm with digital image analysis [27] (Figure 3E), we quantified KRT7 MFI and confirmed the predominant staining in basal-labeled cells (Figure 4A), with no staining either in the luminal benign or tumoral compartments or in the stroma, when compared to the negative control slide (Figure 4B). It was then decided to focus on and quantify the expression of KRT7 expression specifically in the cellular compartment identified with the basal mask.

To assess the prognostic capacity of KRT7 expression in basal cells, this feature was evaluated to determine if it was associated with BCR. Based on Heagerty’s variant to the ROC curve to produce an area under the curve (AUC) for BCR, basal cell KRT7 had an AUC of 0.523 (data not shown). The median KRT7 MFI value was used to dichotomize KRT7 basal cell expression data, but with no significant association with BCR (*p* = 0.365 for median MFI value of 11.95 used as the cut-off for Kaplan–Meier analyses) (Supplementary Figure S6A). MFI values dichotomized by the mean MFI values or categorized by quartiles resulted in no significant association with BCR (data not shown).

To refine our analysis, data were then stratified as quintiles to identify groups most susceptible to predict BCR and to determine the appropriate threshold to dichotomize the basal cell expression level of KRT7. In basal benign epithelial cells, quintile analysis of KRT7 expression revealed that the upper group (>5th quintile or >80th percentile of MFI = 17.56; yellow curve) presented a trend of association with BCR (*p* = 0.123), compared to the other curves (Supplementary Figure S6B). This dichotomization was associated with significant differences in IF patterns, differentiating KRT7-low patient cores with staining mainly in basal cells, from KRT7-high patient cores with staining in basal and supra-basal cells (Figure 4C). Interestingly, supra-basal staining of KRT7 was mostly present on hypertrophic clusters of cells. This hypertrophic pattern (also described in healthy prostate, and similar to urothelial metaplasia) was frequently associated with an atrophic gland pattern and was found in 87.5% of the 64 patients with an MFI >80th percentile (>17.56). Of note, KRT7-high patient cores were not associated with intra-ductal carcinomas features (described in only 4/64 KRT7-high patients, 6.25%).

### 3.4. Prognostic Value of High KRT7 Expression in the Basal Compartment

**Figure 5:**
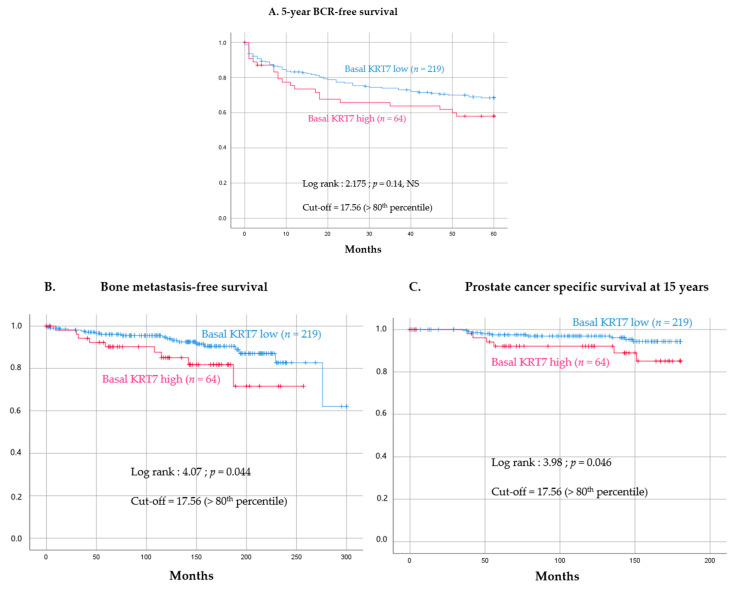
Association of KRT7-high expression (>80th percentile) in basal epithelial cells from benign peri-tumoral glands with survival. (**A**) Association of KRT7-high expression with patient risk of 5year-biochemical recurrence; mean time estimation: 41.2 months (CI 95%: 34.6–47.8) for the KRT7-high vs. 46.3 months (CI 95%: 43.3–49.2) for the KRT-low arm, (**B**) metastasis-free survival; mean time estimation: 216.9 months (CI 95%: 192.9–240.8) for the KRT7-high vs. 268 months (CI 95%: 253.6–282.4) for the KRT-low arm, (**C**) and prostate-cancer specific survival at 15 years; mean time estimation: 167 months (CI 95%: 156.8–177.2) for the KRT7-high vs. 175 months (CI 95%: 171.8–178.4) for the KRT-low arm. These risks were evaluated by Kaplan–Meier analysis coupled with a log-rank test. A *p*-value < 0.05 was considered statistically significant. NS: not significant.

Using this dichotomization cut-off (≤80th and >80th percentile), it was found that KRT7-high expression in the basal compartment from peri-tumoral benign glands was still associated with a non-significant trend towards a higher risk of BCR (log-rank = 2.175, *p* = 0.14) (Figure 5A). Remarkably, KRT7-high expression was significantly associated with an increased risk of developing bone metastasis (28 events, log-rank = 4.07, *p* = 0.044) (Figure 5B) and an increased risk of PC-associated death at 15 years (15 events, log-rank = 3.98, *p* = 0.046) (Figure 5C).

Based on these results, univariate Cox regression analysis was performed for KRT7 high expression in basal cells to test for bone metastasis onset prediction only (no significant differences for BCR prediction, and too few events in PC mortality to perform Cox regression analysis). KRT7 expression dichotomized as quintiles (with KRT7-high defined as >80th percentile) showed an association with increased risk of bone metastasis onset (HR = 2.238, IC 1.002–4.999, *p* = 0.049) (Table 1). Of note, continuous KRT7 values failed to show significance (*p* = 0.397). The multivariate Cox regression analysis (on 28 events) included only the Gleason score (dichotomized as 0 = Gleason Grade Group 1 and 2, and 1 = Gleason Grade Group ≥ 3). KRT7-high expression (>80th percentile) was also found to be statistically significant in a multivariable model (HR = 2.907, *p* = 0.014) and identified as an independent marker to predict the onset of bone metastasis, but with lower predictive capacity than the Gleason score (HR = 27.014, *p* < 0.001) (Table 1).

As a confirmation, when studying the correlation between KRT7-high and Gleason score (as described by previous authors [20]), no correlation was found (Pearson r = −0.009, *p* = 0.877), meaning that KRT7-high immunoreactivity in benign peri-tumoral glands was not systematically correlated with high-risk disease, but independently predictive of MFS. Moreover, the negative predictive value of KRT7-high expression to anticipate bone metastasis onset was 94%, meaning that a KRT7-low expression ≤ 80th was a good predictor for absence of bone metastasis development.

## 4. Discussion

Biomarker validation remains critical to accurately predict metastasis and PC-specific mortality and to be able to discriminate patients who need radical therapy (prostatectomy or radiation) from those who can be safely managed by active surveillance. Much research is now focused on identifying the tumor-initiating cells/castration-tolerant cells that could lead to progression on active surveillance or recurrence after aggressive treatment, and adult stem/progenitor cells are alleged to be those critical cells [30]. KRT7 has been described as a recent novel prostate progenitor cell marker in mice and has been identified in both the basal and luminal cell layers of the mouse prostate, with a preferential localization in ducts and in the proximal prostate [21].

In this study, KRT7 was unexpectedly observed in human prostate tissue, either in autoptic normal glands or in peri-tumoral benign glands. Its staining was mostly found in basal or supra-basal cells from healthy or benign glands, and no staining was present in tumoral luminal cells. Interestingly, this staining in peri-tumoral benign glands was preferentially found in proximal glands (close to the tumor), either in the cohort of low-risk localized PC (*n* = 285) or in the cohort of high-risk PC (i.e., ductal carcinomas, *n* = 16). In poorly differentiated advanced tumors, a global loss of the KRT7 staining was observed in most patients, except for 2/91 with sporadic staining, considered as aberrant. However, in localized PC, it was shown that KRT7 high expression in the basal compartment from peri-tumoral benign glands was associated with shorter bone metastasis-free survival and PC-specific mortality. This observation was independent of the Gleason score to predict MFS. Remarkably, a low KRT7 staining was associated with a 94% negative predictive value for bone metastasis onset.

Overall, our work emphasized that a staining of KRT7 in the prostatic basal compartment (either from normal healthy glands or from peri-tumoral benign glands) was physiological and that its moderate/absent expression could be considered as a marker of good prognosis, conversely to its overexpression in proximal peri-tumoral basal glands, with a loss of staining in almost all tumoral cells.

The identification of KRT7 as a possible prognostic factor only in benign tissue adjacent to the tumor argues in favor of the possible interaction between benign glands and the tumor, with previously published reports showing that tumor-instructed normal tissue or DNA-methylation in the benign tissue could impact the volume and aggressiveness of tumors in PC [31,32]. Moreover, it has been shown by other authors that prostate tumors could interact with the surrounding benign prostate epithelium, by downregulating some biomarkers (i.e., microseminoprotein-beta, MSMB), and that this response could be associated with tumor aggressiveness [33]. We also reported a correlation between the high expression of biomarkers found in the paired adjacent benign epithelium (PUMA/NOXA, WISP1, CD73) and either the stage of the tumor or the shorter BCR-free survival [24,34,35]. Conversely, the increased frequency of other biomarkers (i.e., nuclear p65) in tumoral cells from the same cohort of patients was also correlated with the risk of disease progression [36].

The role of the micro-environment and the stroma has also been suggested with the description of a complex regulation of luminal progenitor cells via interactions between stromal cells surrounding the proximal prostatic ducts and the basal cells [37]. Leclerc et al. showed that the expression of CD73 in the tumor stroma was negatively correlated to p65 biomarker expression in the nuclei of prostate tumor cells [35]. Moreover, high levels of either WISP1 or CD73 were correlated with the density of immunosuppressive CD8+ T cells [34,35,38], thus leading to PC growth, metastasis, and disease progression [34]. Expression of KRT7 has been significantly associated with immune infiltration of tumor immune cells and immunomodulators in other carcinomas [39]. Thus, KRT7-positive benign epithelial cells surrounding prostate tumors may be also involved in functional interactions with immune cells.

Regarding the localization of KRT7 staining in benign prostatic glands, few authors have reported a similar KRT7 staining [29], but the staining of normal prostatic ducts has already been described as physiological [19,40]. In particular, Bassily et al. found a KRT7-positive staining in 97% of the benign atrophic prostatic glands and in some basal and secretory cells in benign prostatic acini [29]. Interestingly, in their study, some entrapped atrophic prostatic glands were intensely stained in a background of negatively stained malignant acini, exactly as observed in the localized PC cohort of 285 patients. The KRT7-positive basal hypertrophy that we described in some healthy glands has been found in 8–10% of autopsy specimens by other authors [41], with association with histological atrophy but no correlation with future risk of PC [42]. Remarkably, this basal hypertrophy/glandular atrophy pattern was found in 87.5% among the 64 patients described as high KRT7 (>80th percentile). The lining epithelium of the prostatic urethra and seminal vesicles was also stained positively in the study of Bassily et al., and KRT7 stained intensely in benign urothelium in 64% of the cases. Urothelial staining was used as a positive control on our radical prostatectomy slides where KRT7 staining was observed in the urethral urothelium, as described previously [40]. Some authors have described basal cell hyperplasia as being associated with squamous or urothelial metaplasia, which is commonly found in prostates and which is known to express KRT7 when extensive [43].

In tumoral cells, a negative staining was observed which is in line with IHC data from the literature, since KRT7 IHC staining (together with KRT20 staining) is usually used to discriminate the origin of a poorly differentiated tumor between KRT7-negative prostatic tumors and KRT7-positive urothelial tumors [29]. Most of the historical studies do not report any immunoreactivity of KRT7 in prostatic carcinomas [29,44], but some authors describe an “aberrant” KRT7 staining on rare individual cells within otherwise nonreactive tumor areas [19]. Raemekers et al. used the same OV-TL 12/30 clone in IF and found only sporadic cells positive for KRT7 in PC tissues, while KRT7 was systematically positive on urothelial bladder carcinomas [19]. They concluded that even if prostate carcinomas were considered normally negative for KRT7, some tumors may exhibit KRT7 reactivity, ranging from a few scattered positive cells via positive tumor areas to homogeneously positive tumors [19]. One could argue that the positivity of staining in IHC depends on the cut-off percentage used for the definition of “true-positive” patients, which could lead to differences in results. Some authors used a 5% cut-off percentage in IHC to eliminate more “false-positive” results and describe no positive staining in PC [44]. In IF, conversely, the discrimination is mostly done with an MFI cut-off which discriminates positive from negative patients.

Other teams have reported a higher KRT7 positivity in PC (10–49.8%) [20,29,45,46]. Indeed, Bassily et al. were able to find KRT7 staining in only 6/59 (10%) of radical prostatectomy specimens from localized PC [29], whereas in Goldstein’s work (using the same OV-TL 12/30 antibody), KRT7 was reactive in 49.8% of PC [20]. However, in their study, most reactive neoplasms had KRT7 staining of fewer than 25% of the cells, with none of the reactive neoplasm exhibiting KRT7 staining in more than 50% of the cells. Moreover, compared to our cohort (285 patients) of localized PC samples with only 10.2% of patients exhibiting a high risk of recurrence (defined by a Gleason score ≥8), the cohort described by Goldstein et al. was mainly composed of high-risk patients (150/225, 66.7%). In the latter, the percentage of KRT7-reactive prostatic carcinomas and the percentage of KRT7-stained cells increased in higher Gleason score PC (from 32% of neoplasms with Gleason score of 6 to 74% of the neoplasms with a Gleason score of 10). The predominant staining pattern in reactive cases was finely granular cytoplasmic staining on rare individual cells in PC with lower Gleason scores and widely dispersed clusters of several reactive cells in PC with higher Gleason scores.

It must be noticed that in these previous studies, no discrimination was done in the quantification of KRT7 staining between tumor and adjacent benign glands, which may explain the discrepancy. The predominance of low-risk patients, in the localized cohort we used, could also explain the lower or absent KRT7 staining in tumoral cells. Overall, Goldstein et al. concluded that an extensive KRT7 reactivity should not be encountered in a PC, but that a sparse KRT7 staining should not be an unexpected finding, especially in neoplasms with higher Gleason scores [20]. This result, however, differs from the results of other studies in which the authors noted that reactivity of KRT7 (and KRT20) was similar among PC of different Gleason scores [45,47,48]. Similarly, we found no significant correlation between a high KRT7 expression and the Gleason score on the localized PC cohort and only an aberrant staining in patients with advanced PC (2.2%). Moreover, regarding another aggressive feature such as intra-ductal carcinoma, we found no association between a high KRT7 expression and this pattern in our cohort of 285 patients (6.25% of intra-ductal carcinoma in KRT7-high patients). Of note, our results were confirmed using both a mouse or a rabbit antibody, and with two different staining techniques (IF/IHC) reinforcing the accuracy of this finding.

Regarding advanced PC, access to available tissue cohorts (either prostatic tissue or metastases) is rare. Bassily et al. described an autopsy cohort of 10 metastatic specimens (from patients who died from CR PC), with only a few patients showing a focally positive KRT7 staining, similar to the aberrant staining found in 2/91 patients from our TURP-TMA cohort [29]. These differences between metastatic state and localized cancer can somehow be linked to cell plasticity, which could explain the loss of some markers in tumoral cells compared to benign glands [49]. Interestingly, some authors have emphasized the fact that ADT used in metastatic PC to inhibit the androgen receptor (AR) activation, could promote a lineage plasticity with modifications in staining for prostate biomarkers [50]. Some stem cell markers were found as highly expressed in AR-negative basal epithelial cells from benign prostate tissue and low in luminal tumoral cells, in a similar pattern to the one we observed for KRT7. Further studies are warranted to test whether this mixed basal/luminal identity is a common early step in CRPC lineage plasticity that enables subsequent reprogramming to establish neuro-endocrine PC or other cell lineage states observed in advanced tumors.

To date, we are the first to describe the KRT7 staining in PC as a predictive biomarker. Indeed, we have shown that the basal staining in peritumoral benign glands was highly predictive of bone-metastasis appearance, PC-specific mortality but not BCR. Low KRT7 staining was indicative of a good prognosis and was associated with a negative predictive value of 94% for bone metastasis onset. The intermediate clinical endpoints for prostate cancer (ICECaP) consortium showed in 2017 that MFS was a surrogate of overall survival (OS) in non-metastatic patients, with a significant risk of death from PC in these patients [51]. As a confirmation, a publication from 2021 has highlighted the fact that only MFS, and not BCR, was a strong surrogate marker of PC-specific mortality since the development of bone metastasis is linked to the castration-resistant state when ADT is initiated in these patients [12]. The authors concluded that this endpoint should be the one on which new treatments aiming to improve OS are approved and adopted, and that ongoing investigations and incorporation of biomarkers are needed to identify potential surrogate endpoints that can be assessed earlier than MFS. Indeed, assessment of metastasis requires mature cohorts with matched histological or molecular data and long-term follow-up, criteria that were fulfilled by our cohort of 285 patients.

No previous study had identified KRT7 as a putative prognostic biomarker in PC, and only few conflictual data described an association between the Gleason score and percentage of KRT7 positivity [20,52]. However, its overexpression alone or in combination (E-cadherin) in several other cancer types has been reported to predict a poor prognosis [15,16,17,18]. On the other hand, high KRT7 expression was associated with a better prognosis in renal cell carcinoma, as an example [53,54]. These conflicting data and the correlation that we found on a monocentric cohort requires to be confirmed on a larger external and multicentric cohort.

Some authors also found that the localization of KRT7 staining could influence the correlation with survival. In colorectal cancer, KRT7 is usually not expressed in the normal colonic epithelium or localized colorectal cancer (using the same OV-TL 12/30 antibody) but was found in some lymph node metastatic cells [17]. In this study, only the expression of KRT7 in lymph nodes was correlated with shorter overall survival and the presence of distant metastases at diagnosis, whereas expression of KRT7 in the primary tumor did not correlate with survival.

Together, these observations emphasize the major but complex and tissue-specific functional implications of KRT7 function in cancer progression. Roles have been described for keratins other than KRT7 in the immune system and inflammation, DNA damage response and resistance to apoptosis, or apico-basal polarization [55,56]. Functional enrichment analyses and GSEA indicated that KRT7 might be involved in the regulation of the p53 pathway in pancreatic adenocarcinoma [39]. KRT7 was also positively correlated with keratin-8 (KRT8) expression in an intra-cellular gene-gene correlation, with KRT8 having been identified as a pan-cancer early biomarker in a multi-scale integrated analysis [57]. Another cytokeratin, described as a putative heterodimer partner of KRT7, i.e., KRT17, has also been shown to be significantly lower in aggressive cancers (diffuse gastric cancer), similar to the loss of KRT7 staining that we observed in tumoral prostatic glands [58]. In this study, the downregulation of KRT17 induced E-cadherin loss, epithelial-mesenchymal transition (EMT) changes, and metastatic behavior of tumoral cells. Mechanistically, the loss of intermediate filaments of KRT17 induced reorganization of the cytoskeleton further activated YAP signaling, and increased IL-6 expression, ultimately leading to the enhanced metastasis ability. The putative role of KRT7 as a partner of KRT17 could provide a new insight into the role of aberrant intermediate filaments in malignancy and the onset of metastasis. KRT7 could act as a biomarker of a particular state, more than a marker of a distinct cellular population per se. The recent publication of a single-cell analysis of human primary prostate cancer uncovered heterogeneous cellular states in prostate epithelial cells from both cancer areas and paired-matched normal tissue adjacent to the tumor [59]. These results suggested a cellular plasticity, and either KRT17 or KRT7 were described as markers of particular cellular states (basal and club cells, respectively), thus providing some insight into a putative functional role of KRT7 [59].

Together, these studies put into perspective the relevance of our results, specifically found in benign tissue, where the risk of metastasis onset can potentially be determined on prostatic biopsy specimens containing benign tissue. These benign cores represent the majority of the cores of patients recommended for active surveillance, where only 1–2 tumoral cores of indolent PC (i.e., Gleason 6) are found. With a negative predictive value of 94%, the low KRT7 expression on these benign cores could confidently indicate a very low risk of metastasis onset. In addition, keratins are widely used to detect circulating tumor cells in the blood and monitor prognosis [56]. Notably soluble protein fragments of KRT7 can be detected in the circulation of cancer patients or in other fluids such as ascites [56], with the recent assessment of KRT7-positive prostatic cells in the prostatic fluid expressed during robot-assisted radical prostatectomy [60], which could lead to further liquid biopsy (circulating tumor cells) assessments in PC patients, either in blood, prostatic fluid, or urine.

## 5. Conclusions

Prostate carcinomas are considered KRT7-negative tumors and we confirmed that KRT7 expression was lost in tumoral cells and only sporadically found in advanced tumors. Conversely, KRT7 expression was found in the basal and supra-basal compartment from proximal benign glands adjacent to the tumors, and its high expression was independently correlated with MFS and CSS. The KRT7 expression on benign cores from prostate biopsies could help segregate patients and recommend low-KRT7 patients for active surveillance, while proposing radical treatment to high-KRT7 patients (MFI of KRT7 > 80th percentile) due to a higher risk of the onset of bone metastasis. Further validation on larger cohorts is warranted and ongoing.

## Figures and Tables

**Table 1:** Univariate and multivariate Cox regression analyses of Gleason score and KRT7 expression (either continuous value or dichotomized with quintiles) in basal cells to predict bone metastasis free survival.

Type of Analyses	Univariate		Multivariate	
	**HR [95% CI]**	***p*** **-Value**	**HR [95% CI]**	***p*** **-Value**
Age at diagnosis	0.99 [0.926–1.059]	0.777	-	-
cTNM	1.304 [0.507–3.355]	0.581	-	-
Gleason score 0–1	**21.407** [8.663–52.9]	**<0.001**	**27.014** [10.13–72.05]	**<0.001**
KRT7 MFI expression in basal cells with continuous value	1.021 [0.973–1.073]	0.397	-	-
KRT7 MFI expression in basal cells dichotomized with quintiles (high-KRT7 defined as >80th percentile)	**2.238 [1.002–4.999]**	**0.049**	**2.907 [1.229–6.875]**	**0.014**

Abbreviations: CI = confidence interval; HR = hazard ratio; cTNM = clinical TumorNodesMetastasis. Gleason score dichotomized as 0 = Gleason Grade Group 1 and 2, and 1 = Gleason Grade Group ≥ 3. Significant results (*p*-value < 0.05) are indicated by bold numbers and results not included are indicated by -.

## Data Availability

Data presented is contained within the article; for additional information, data sets are also available upon request from the corresponding author.

## Supplementary Materials

The following are available online at https://www.mdpi.com/article/10.3390/cancers14071623/s1, Figure S1: Validation of KRT7 mouse OV-TL 12/30 and rabbit SP52 monoclonal antibody specificity in prostate cancer cell lines and their derivatives and Jurkat T lymphoma cell lines, Figure S2: Whole Western blots of KRT7 using two antibodies in PC cell lines and Jurkat hematological cell line, Figure S3: KRT7 expression in normal healthy prostate, Figure S4: Additional examples of KRT7 staining by immunohistochemistry (IHC) using KRT7 rabbit SP52 clone on FFPE prostatic tissues section of radical prostatectomy from 2 other patients (Patient #2 and Patient #3) with prostatic ductal carcinoma, Figure S5: Detection of KRT7 expression by IHC in TURP cores. IHC assay with KRT7 staining using DAB kit detection on FFPE-cores from the TURP-TMA, *n* = 91 patients (clone OV-TL 12/30 mouse), Figure S6: Impact of KRT7 basal expression on patient risk of biochemical recurrence (BCR) evaluated by Kaplan–Meier analysis coupled with a log-rank test, using various cut-offs of mean fluorescence intensity (MFI); Table S1: Clinical and pathological characteristics of the 285 patients who underwent radical prostatectomy.

## References

[1] Siegel R.L., Miller K.D., Jemal A. (2020). Cancer Statistics, 2020. CA Cancer J. Clin..

[2] Karantanos T., Corn P.G., Thompson T.C. (2013). Prostate Cancer Progression after Androgen Deprivation Therapy: Mechanisms of Castrate Resistance and Novel Therapeutic Approaches. Oncogene.

[3] Zhou C.K., Check D.P., Lortet-Tieulent J., Laversanne M., Jemal A., Ferlay J., Bray F., Cook M.B., Devesa S.S. (2016). Prostate Cancer Incidence in 43 Populations Worldwide: An Analysis of Time Trends Overall and by Age Group. Int. J. Cancer.

[4] Beesley L.J., Morgan T.M., Spratt D.E., Singhal U., Feng F.Y., Furgal A.C., Jackson W.C., Daignault S., Taylor J.M.G. (2019). Individual and Population Comparisons of Surgery and Radiotherapy Outcomes in Prostate Cancer Using Bayesian Multistate Models. JAMA Netw. Open.

[5] Albertsen P.C., Hanley J.A., Penson D.F., Barrows G., Fine J. (2007). 13-Year Outcomes Following Treatment for Clinically Localized Prostate Cancer in a Population Based Cohort. J. Urol..

[6] Jackson W.C., Suresh K., Tumati V., Allen S.G., Dess R.T., Salami S.S., George A., Kaffenberger S.D., Miller D.C., Hearn J.W.D. (2018). Intermediate Endpoints After Postprostatectomy Radiotherapy: 5-Year Distant Metastasis to Predict Overall Survival. Eur. Urol..

[7] D’Amico A.V., Whittington R., Malkowicz S.B., Schultz D., Blank K., Broderick G.A., Tomaszewski J.E., Renshaw A.A., Kaplan I., Beard C.J. (1998). Biochemical Outcome after Radical Prostatectomy, External Beam Radiation Therapy, or Interstitial Radiation Therapy for Clinically Localized Prostate Cancer. JAMA.

[8] Kattan M.W., Eastham J.A., Stapleton A.M., Wheeler T.M., Scardino P.T. (1998). A Preoperative Nomogram for Disease Recurrence Following Radical Prostatectomy for Prostate Cancer. J. Natl. Cancer Inst..

[9] Cooperberg M.R., Pasta D.J., Elkin E.P., Litwin M.S., Latini D.M., Du Chane J., Carroll P.R. (2005). The University of California, San Francisco Cancer of the Prostate Risk Assessment Score: A Straightforward and Reliable Preoperative Predictor of Disease Recurrence after Radical Prostatectomy. J. Urol..

[10] Dess R.T., Suresh K., Zelefsky M.J., Freedland S.J., Mahal B.A., Cooperberg M.R., Davis B.J., Horwitz E.M., Terris M.K., Amling C.L. (2020). Development and Validation of a Clinical Prognostic Stage Group System for Nonmetastatic Prostate Cancer Using Disease-Specific Mortality Results From the International Staging Collaboration for Cancer of the Prostate. JAMA Oncol..

[11] Hartman H.E., Jackson W.C. (2020). Surrogate Endpoints in Localized Prostate Cancer. Cancer J..

[12] Gharzai L.A., Jiang R., Wallington D., Jones G., Birer S., Jairath N., Jaworski E.M., McFarlane M.R., Mahal B.A., Nguyen P.L. (2021). Intermediate Clinical Endpoints for Surrogacy in Localised Prostate Cancer: An Aggregate Meta-Analysis. Lancet Oncol..

[13] Mazzone E., Gandaglia G., Ploussard G., Marra G., Valerio M., Campi R., Mari A., Minervini A., Serni S., Moschini M. (2021). Risk Stratification of Patients Candidate to Radical Prostatectomy Based on Clinical and Multiparametric Magnetic Resonance Imaging Parameters: Development and External Validation of Novel Risk Groups. Eur. Urol..

[14] Trompetter M., Smedts F., Van der Wijk J., Schoots C., de Jong H.-J., Hopman A., De la Rosette J. (2008). Keratin Profiling in the Developing Human Prostate. A Different Approach to Understanding Epithelial Lineage. Anticancer Res..

[15] Oue N., Noguchi T., Anami K., Kitano S., Sakamoto N., Sentani K., Uraoka N., Aoyagi K., Yoshida T., Sasaki H. (2012). Cytokeratin 7 Is a Predictive Marker for Survival in Patients with Esophageal Squamous Cell Carcinoma. Ann. Surg. Oncol..

[16] Yang J. (2020). Identification of Novel Biomarkers, MUC5AC, MUC1, KRT7, GAPDH, CD44 for Gastric Cancer. Med. Oncol..

[17] Czapiewski P., Bobowicz M., Pęksa R., Skrzypski M., Gorczyński A., Szczepańska-Michalska K., Korwat A., Jankowski M., Zegarski W., Szulgo-Paczkowska A. (2016). Keratin 7 Expression in Lymph Node Metastases but Not in the Primary Tumour Correlates with Distant Metastases and Poor Prognosis in Colon Carcinoma. Pol. J. Pathol..

[18] Communal L., Roy N., Cahuzac M., Rahimi K., Köbel M., Provencher D.M., Mes-Masson A.-M. (2021). A Keratin 7 and E-Cadherin Signature Is Highly Predictive of Tubo-Ovarian High-Grade Serous Carcinoma Prognosis. Int. J. Mol. Sci..

[19] Ramaekers F., van Niekerk C., Poels L., Schaafsma E., Huijsmans A., Robben H., Schaart G., Vooijs P. (1990). Use of Monoclonal Antibodies to Keratin 7 in the Differential Diagnosis of Adenocarcinomas. Am. J. Pathol..

[20] Goldstein N.S. (2002). Immunophenotypic Characterization of 225 Prostate Adenocarcinomas with Intermediate or High Gleason Scores. Am. J. Clin. Pathol..

[21] Ceder J.A., Aalders T.W., Schalken J.A. (2017). Label Retention and Stem Cell Marker Expression in the Developing and Adult Prostate Identifies Basal and Luminal Epithelial Stem Cell Subpopulations. Stem Cell Res. Ther..

[22] Sackmann Sala L., Boutillon F., Menara G., De Goyon-Pélard A., Leprévost M., Codzamanian J., Lister N., Pencik J., Clark A., Cagnard N. (2017). A Rare Castration-Resistant Progenitor Cell Population Is Highly Enriched in Pten-Null Prostate Tumours. J. Pathol..

[23] Borziak K., Finkelstein J. (2021). Comparative Analysis of Public Data Sets to Identify Stemness Markers That Differentiate Liver Cancer Stem Cells. Stud. Health Technol. Inform..

[24] Clairefond S., Péant B., Ouellet V., Barrès V., Tian Z., Trudel D., Karakiewicz P.I., Mes-Masson A.-M., Saad F. (2020). PUMA and NOXA Expression in Tumor-Associated Benign Prostatic Epithelial Cells Are Predictive of Prostate Cancer Biochemical Recurrence. Cancers.

[25] Clairefond S., Ouellet V., Péant B., Barrès V., Karakiewicz P.I., Mes-Masson A.-M., Saad F. (2021). Expression of ERBB Family Members as Predictive Markers of Prostate Cancer Progression and Mortality. Cancers.

[26] Zietarska M., Madore J., Diallo J.-S., Delvoye N., Saad F., Provencher D., Mes-Masson A.-M. (2010). A Novel Method of Cell Embedding for Tissue Microarrays. Histopathology.

[27] Labouba I., Le Page C., Communal L., Kristessen T., You X., Péant B., Barrès V., Gannon P.O., Mes-Masson A.-M., Saad F. (2015). Potential Cross-Talk between Alternative and Classical NF-ΚB Pathways in Prostate Cancer Tissues as Measured by a Multi-Staining Immunofluorescence Co-Localization Assay. PLoS ONE.

[28] Strand D.W., Goldstein A.S. (2015). The Many Ways to Make a Luminal Cell and a Prostate Cancer Cell. Endocr. Relat. Cancer.

[29] Bassily N.H., Vallorosi C.J., Akdas G., Montie J.E., Rubin M.A. (2000). Coordinate Expression of Cytokeratins 7 and 20 in Prostate Adenocarcinoma and Bladder Urothelial Carcinoma. Am. J. Clin. Pathol..

[30] Baures M., Dariane C., Tika E., Puig Lombardi E., Barry Delongchamps N., Blanpain C., Guidotti J.-E., Goffin V. (2022). Prostate Luminal Progenitor Cells: From Mouse to Human, from Health to Disease. Nat. Rev. Urol..

[31] Adamo H.H., Strömvall K., Nilsson M., Halin Bergström S., Bergh A. (2015). Adaptive (TINT) Changes in the Tumor Bearing Organ Are Related to Prostate Tumor Size and Aggressiveness. PLoS ONE.

[32] Yang B., Etheridge T., McCormick J., Schultz A., Khemees T.A., Damaschke N., Leverson G., Woo K., Sonn G.A., Klein E.A. (2019). Validation of an Epigenetic Field of Susceptibility to Detect Significant Prostate Cancer from Non-Tumor Biopsies. Clin. Epigenetics.

[33] Bergström S.H., Järemo H., Nilsson M., Adamo H.H., Bergh A. (2018). Prostate Tumors Downregulate Microseminoprotein-Beta (MSMB) in the Surrounding Benign Prostate Epithelium and This Response Is Associated with Tumor Aggressiveness. Prostate.

[34] Gaudreau P.-O., Clairefond S., Class C.A., Boulay P.-L., Chrobak P., Allard B., Azzi F., Pommey S., Do K.-A., Saad F. (2019). WISP1 Is Associated to Advanced Disease, EMT and an Inflamed Tumor Microenvironment in Multiple Solid Tumors. Oncoimmunology.

[35] Leclerc B.G., Charlebois R., Chouinard G., Allard B., Pommey S., Saad F., Stagg J. (2016). CD73 Expression Is an Independent Prognostic Factor in Prostate Cancer. Clin. Cancer Res..

[36] Grosset A.-A., Ouellet V., Caron C., Fragoso G., Barrès V., Delvoye N., Latour M., Aprikian A., Bergeron A., Chevalier S. (2019). Validation of the Prognostic Value of NF-ΚB P65 in Prostate Cancer: A Retrospective Study Using a Large Multi-Institutional Cohort of the Canadian Prostate Cancer Biomarker Network. PLoS Med..

[37] Wei X., Zhang L., Zhou Z., Kwon O.-J., Zhang Y., Nguyen H., Dumpit R., True L., Nelson P., Dong B. (2019). Spatially Restricted Stromal Wnt Signaling Restrains Prostate Epithelial Progenitor Growth through Direct and Indirect Mechanisms. Cell Stem Cell.

[38] Shafer-Weaver K.A., Anderson M.J., Stagliano K., Malyguine A., Greenberg N.M., Hurwitz A.A. (2009). Cutting Edge: Tumor-Specific CD8+ T Cells Infiltrating Prostatic Tumors Are Induced to Become Suppressor Cells. J. Immunol..

[39] Li Y., Su Z., Wei B., Liang Z. (2021). KRT7 Overexpression Is Associated with Poor Prognosis and Immune Cell Infiltration in Patients with Pancreatic Adenocarcinoma. Int. J. Gen. Med..

[40] Schaafsma H.E., Ramaekers F.C., van Muijen G.N., Ooms E.C., Ruiter D.J. (1989). Distribution of Cytokeratin Polypeptides in Epithelia of the Adult Human Urinary Tract. Histochemistry.

[41] Thorson P., Swanson P.E., Vollmer R.T., Humphrey P.A. (2003). Basal Cell Hyperplasia in the Peripheral Zone of the Prostate. Mod. Pathol..

[42] Freitas D., Andriole G.L., Freedland S.J., Neto B.S., Moreira D.M. (2019). Baseline Basal Cell Hyperplasia Is Not Associated with Baseline Lower Urinary Tract Symptoms, Baseline Clinical Prostatitis or Prostate Cancer in Repeat Biopsies. Urology.

[43] Henry G., Malewska A., Mauck R., Gahan J., Hutchinson R., Torrealba J., Francis F., Roehrborn C., Strand D. (2017). Molecular Pathogenesis of Human Prostate Basal Cell Hyperplasia. Prostate.

[44] Chu P., Wu E., Weiss L.M. (2000). Cytokeratin 7 and Cytokeratin 20 Expression in Epithelial Neoplasms: A Survey of 435 Cases. Mod. Pathol..

[45] Genega E.M., Hutchinson B., Reuter V.E., Gaudin P.B. (2000). Immunophenotype of High-Grade Prostatic Adenocarcinoma and Urothelial Carcinoma. Mod. Pathol..

[46] Gheitasi R., Sadeghi E., Jafari M. (2021). Comparison of Immunohistochemistry Expression of CK7, HMWK and PSA in High-Grade Prostatic Adenocarcinoma and Bladder Transitional Cell Carcinoma. Iran. J. Pathol..

[47] Cheville J.C., Dundore P.A., Bostwick D.G., Lieber M.M., Batts K.P., Sebo T.J., Farrow G.M. (1998). Transitional Cell Carcinoma of the Prostate: Clinicopathologic Study of 50 Cases. Cancer.

[48] Nagle R.B., Brawer M.K., Kittelson J., Clark V. (1991). Phenotypic Relationships of Prostatic Intraepithelial Neoplasia to Invasive Prostatic Carcinoma. Am. J. Pathol..

[49] Zhang B., Ci X., Tao R., Ni J.J., Xuan X., King J.L., Xia S., Li Y., Frierson H.F., Lee D.-K. (2020). Klf5 Acetylation Regulates Luminal Differentiation of Basal Progenitors in Prostate Development and Regeneration. Nat. Commun..

[50] Che M., Chaturvedi A., Munro S.A., Pitzen S.P., Ling A., Zhang W., Mentzer J., Ku S.-Y., Puca L., Zhu Y. (2021). Opposing Transcriptional Programs of KLF5 and AR Emerge during Therapy for Advanced Prostate Cancer. Nat. Commun..

[51] Xie W., Regan M.M., Buyse M., Halabi S., Kantoff P.W., Sartor O., Soule H., Clarke N.W., Collette L., Dignam J.J. (2017). Metastasis-Free Survival Is a Strong Surrogate of Overall Survival in Localized Prostate Cancer. J. Clin. Oncol..

[52] Kotliar S.N., Wood C.G., Schaeffer A.J., Oyasu R. (1995). Transitional Cell Carcinoma Exhibiting Clear Cell Features. A Differential Diagnosis for Clear Cell Adenocarcinoma of the Urinary Tract. Arch. Pathol. Lab. Med..

[53] Polifka I., Agaimy A., Herrmann E., Spath V., Trojan L., Stöckle M., Becker F., Ströbel P., Wülfing C., Schrader A.J. (2019). High Proliferation Rate and TNM Stage but Not Histomorphological Subtype Are Independent Prognostic Markers for Overall Survival in Papillary Renal Cell Carcinoma. Hum. Pathol..

[54] Mertz K.D., Demichelis F., Sboner A., Hirsch M.S., Dal Cin P., Struckmann K., Storz M., Scherrer S., Schmid D.M., Strebel R.T. (2008). Association of Cytokeratin 7 and 19 Expression with Genomic Stability and Favorable Prognosis in Clear Cell Renal Cell Cancer. Int. J. Cancer.

[55] Sharma P., Alsharif S., Fallatah A., Chung B.M. (2019). Intermediate Filaments as Effectors of Cancer Development and Metastasis: A Focus on Keratins, Vimentin, and Nestin. Cells.

[56] Werner S., Keller L., Pantel K. (2020). Epithelial Keratins: Biology and Implications as Diagnostic Markers for Liquid Biopsies. Mol. Aspects Med..

[57] Scott M.K.D., Ozawa M.G., Chu P., Limaye M., Nair V.S., Schaffert S., Koong A.C., West R., Khatri P. (2021). A Multi-Scale Integrated Analysis Identifies KRT8 as a Pan-Cancer Early Biomarker. Pac. Symp. Biocomput..

[58] Li M., Rao X., Cui Y., Zhang L., Li X., Wang B., Zheng Y., Teng L., Zhou T., Zhuo W. (2021). The Keratin 17/YAP/IL6 Axis Contributes to E-Cadherin Loss and Aggressiveness of Diffuse Gastric Cancer. Oncogene.

[59] Song H., Weinstein H.N.W., Allegakoen P., Wadsworth M.H., Xie J., Yang H., Castro E.A., Lu K.L., Stohr B.A., Feng F.Y. (2022). Single-Cell Analysis of Human Primary Prostate Cancer Reveals the Heterogeneity of Tumor-Associated Epithelial Cell States. Nat. Commun..

[60] Lyon T.D., Henry M.R., Shah P.H., Boorjian S.A., Tollefson M.K., Frank I. (2021). Development of a Technique for Evaluating the Presence of Malignant Cells in Prostatic Fluid during Robotic Prostatectomy. Urol. Oncol..

