# Supplementary material for: High Keratin-7 Expression in Benign Peri-Tumoral Prostatic Glands Is Predictive of Bone Metastasis Onset and Prostate Cancer-Specific Mortality
Source: Cancers (Basel). 2022 Mar 23;14(7):1623. doi: 10.3390/cancers14071623 (PMC8997075; doi:10.3390/cancers14071623)
Supplement: Supplementary file 1 [file cancers-14-01623-s001.zip › cancers-1652110-supplementary.pdf]

cancers

MDPI

Supplementary Materials: High Keratin-7 Expression in Benign Peri-Tumoral Prostatic Glands Is Predictive of Bone Metastasis Onset and Prostate Cancer-Specific Mortality

Charles Dariane, Sylvie Clairefond, Benjamin Péant, Laudine Communal, Zhe Thian, Véronique Ouellet, Dominique Trudel, Nazim Benzerdjeb, Feryel Azzi, Arnaud Méjean, Marc-Olivier Timsit, Manon Baurès, Jacques-Emmanuel Guidotti, Vincent Goffin, Pierre I. Karakiewicz, Anne-Marie Mes-Masson and Fred Saad

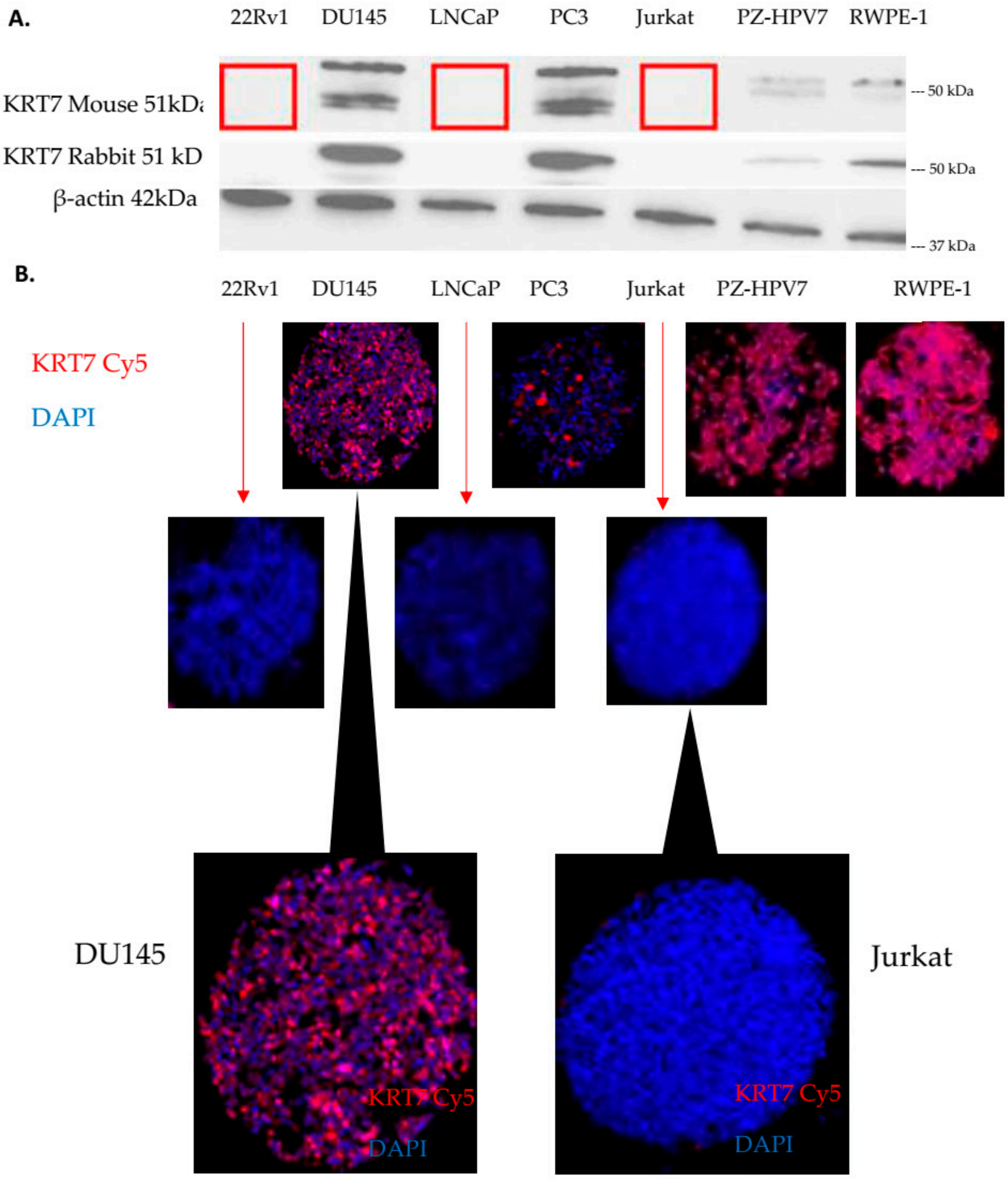

Figure S1. Validation of KRT7 mouse OV-TL 12/30 and rabbit SP52 monoclonal antibody specificity in prostate cancer cell lines and their derivatives and Jurkat T lymphoma cell lines. (**A**) Expression of KRT7 in tested cell lines. Expression was assessed by Western blot, with positive prostatic cell lines (DU145, PC3, PZ-HPV7 and RWPE-1) and negative hematologic and hormone-sensitive PC

cell lines (Jurkat T lymphoma and 22 Rv1, LNCaP). β-actin served as loading control. (**B**) Detection of KRT7 expression by immunofluorescence in paraffin-embedded cell pellets using KRT7 mouse OV-TL 12/30 monoclonal antibody. Magnification of DU145 (positive control) and Jurkat (negative control) cell pellets. DAPI in blue and KRT7 in red (Cy5).

A.

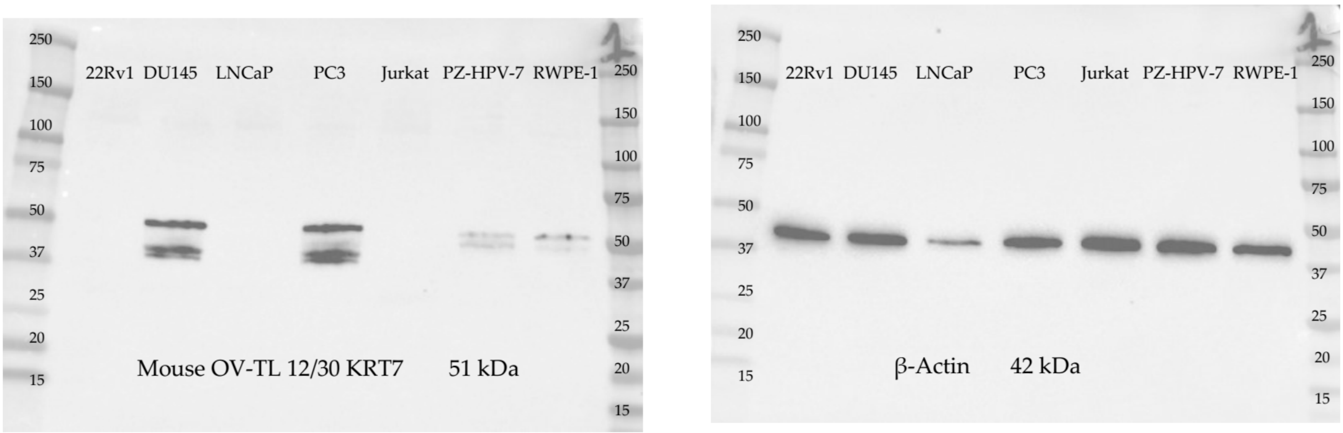

B.

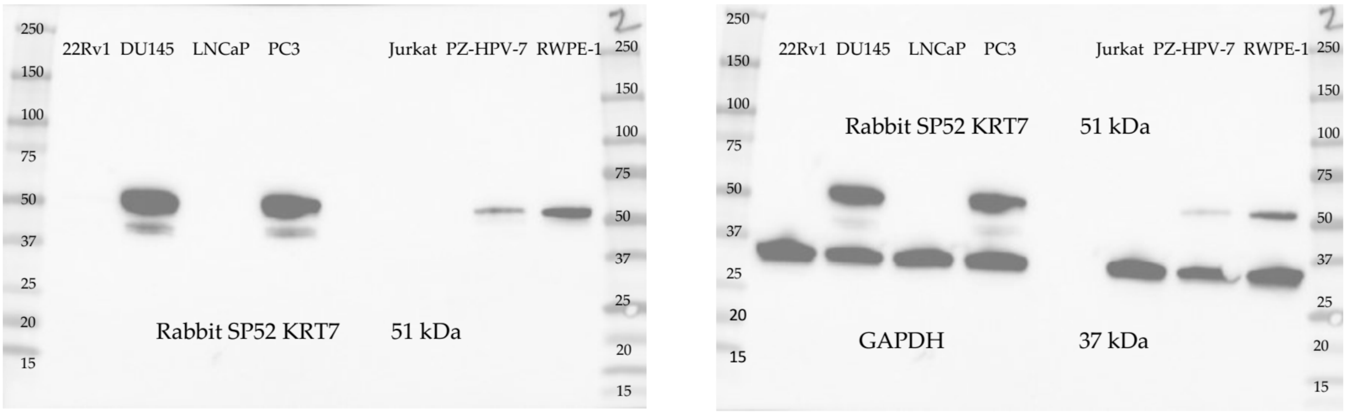

C.

	22Rv1	DU145	LNCaP	PC3	Jurkat	PZ-HPV-7	RWPE-1
Mouse KRT7	0.008	2.42	0.033	2.61	0.01	0.38	0.69
Rabbit KRT7	0.01	3.54	0.007	2.3	0.009	0.24	0.92

Figure S2. Whole Western blots of KRT7 using two antibodies in PC cell lines and Jurkat hematological cell line. (**A**) Detection of mouse OV-TL 12/30 KRT7 in whole cell lysates, with β-actin as a control. (**B**) Detection of rabbit SP52 KRT7 in whole cell lysates, with GAPDH as a control on the same membrane. (**C**) Intensity ratio of KRT7 for each cell line, normalized with either β-actin or GAPDH.

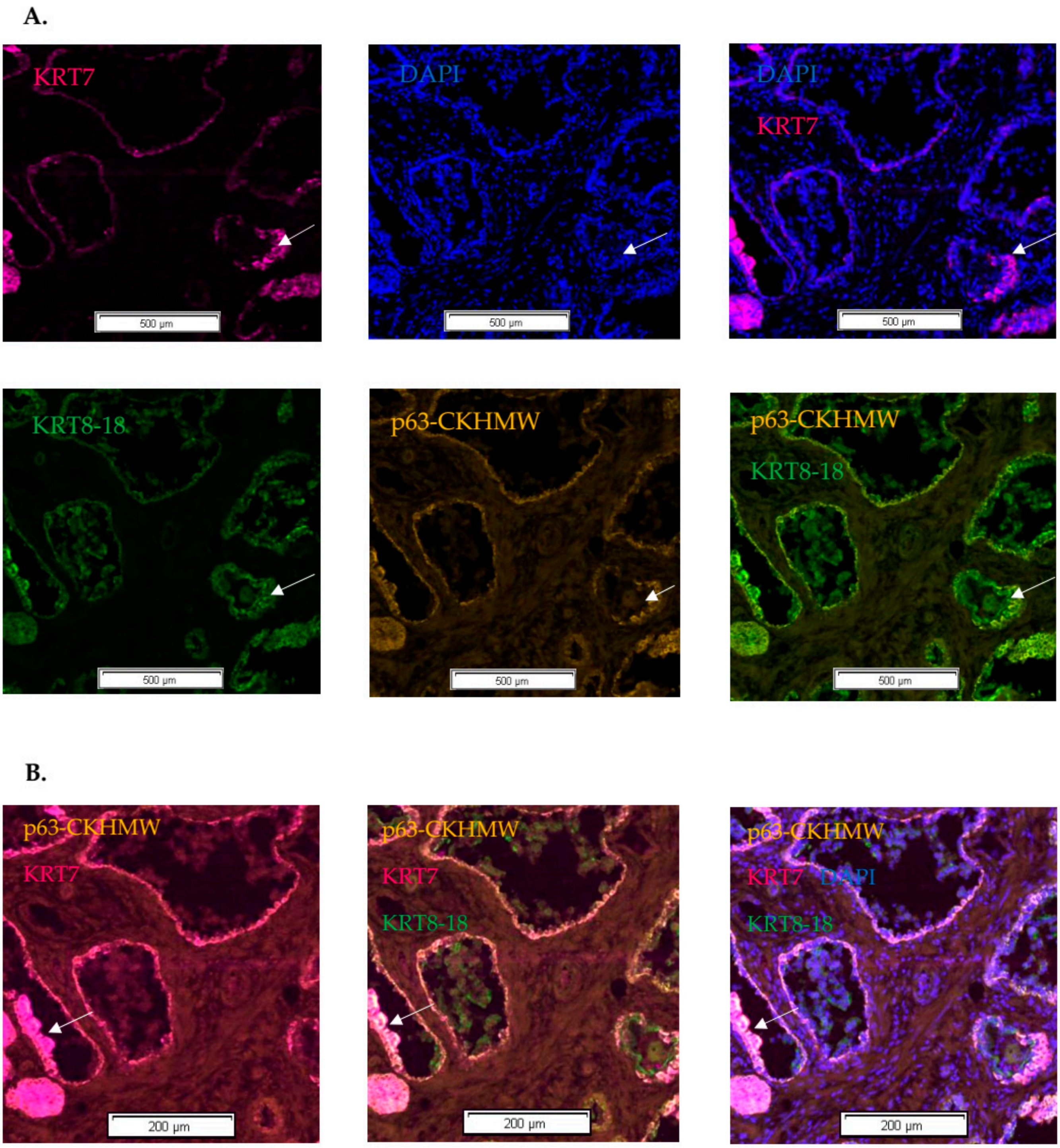

Figure S3. KRT7 expression in normal healthy prostate. (**A**) Multiplex IF co-staining on normal prostate from an autopsy of an 18-year-old patient. Separate staining to discriminate nuclei (DAPI blue), epithelial cell mask (KRT8/18, FITC-488 green), benign glands (p63/CKHMW, TRITC-546 yellow) and the studied biomarker KRT7 (Cy7 pink) with the OV-TL 12/30 clone [Cy7-AF750]. (**B**) KRT7 expression in basal cells. Merged multi-staining IF with KRT7 expression in basal cells. Of interest, some glands presented a basal hypertrophy (arrows).

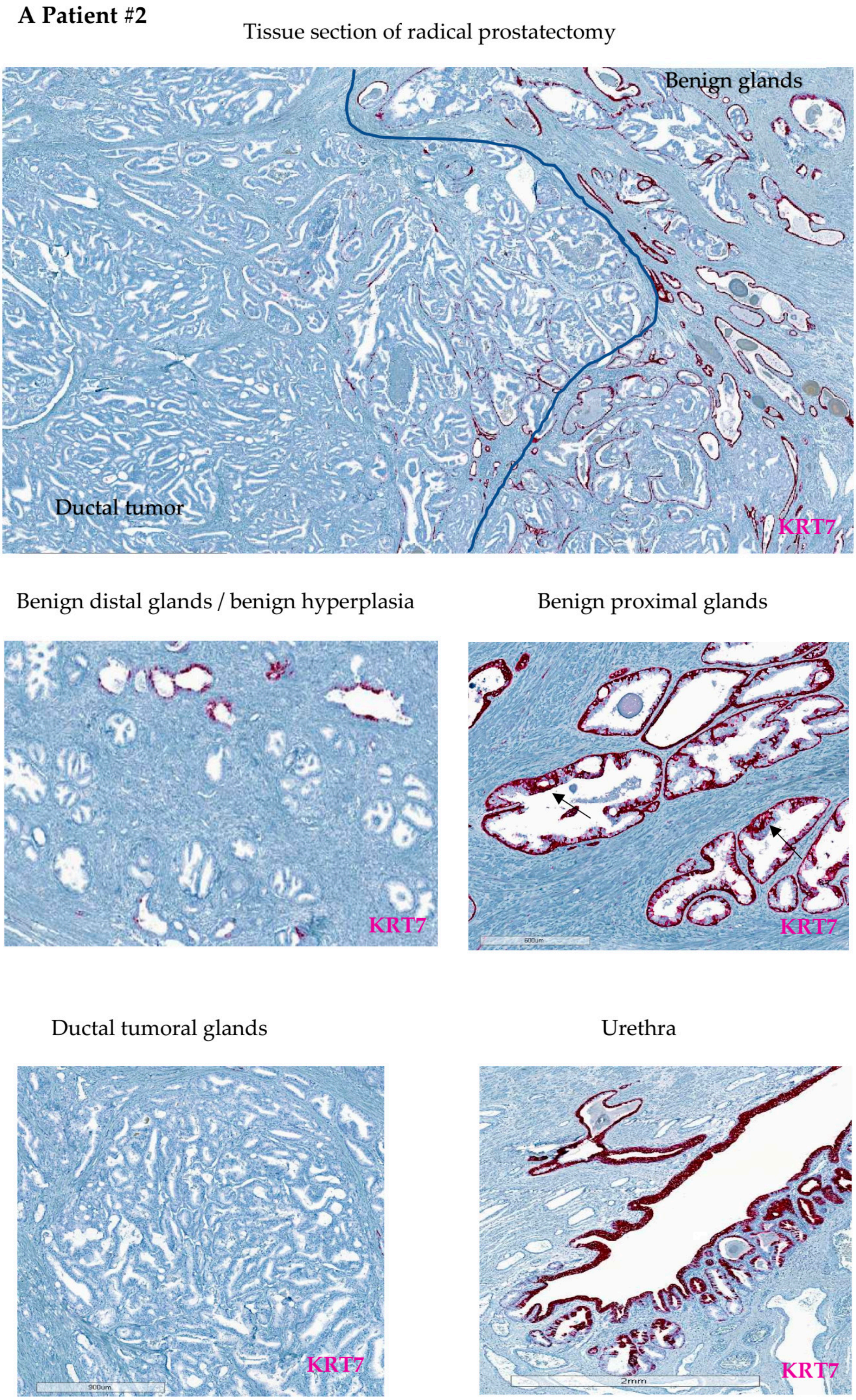
A Patient #2
Tissue section of radical prostatectomy
Benign glands
Ductal tumor
KRT7
Benign distal glands / benign hyperplasia
Benign proximal glands
KRT7
600um
KRT7
Ductal tumoral glands
Urethra
900um
KRT7
2mm
KRT7

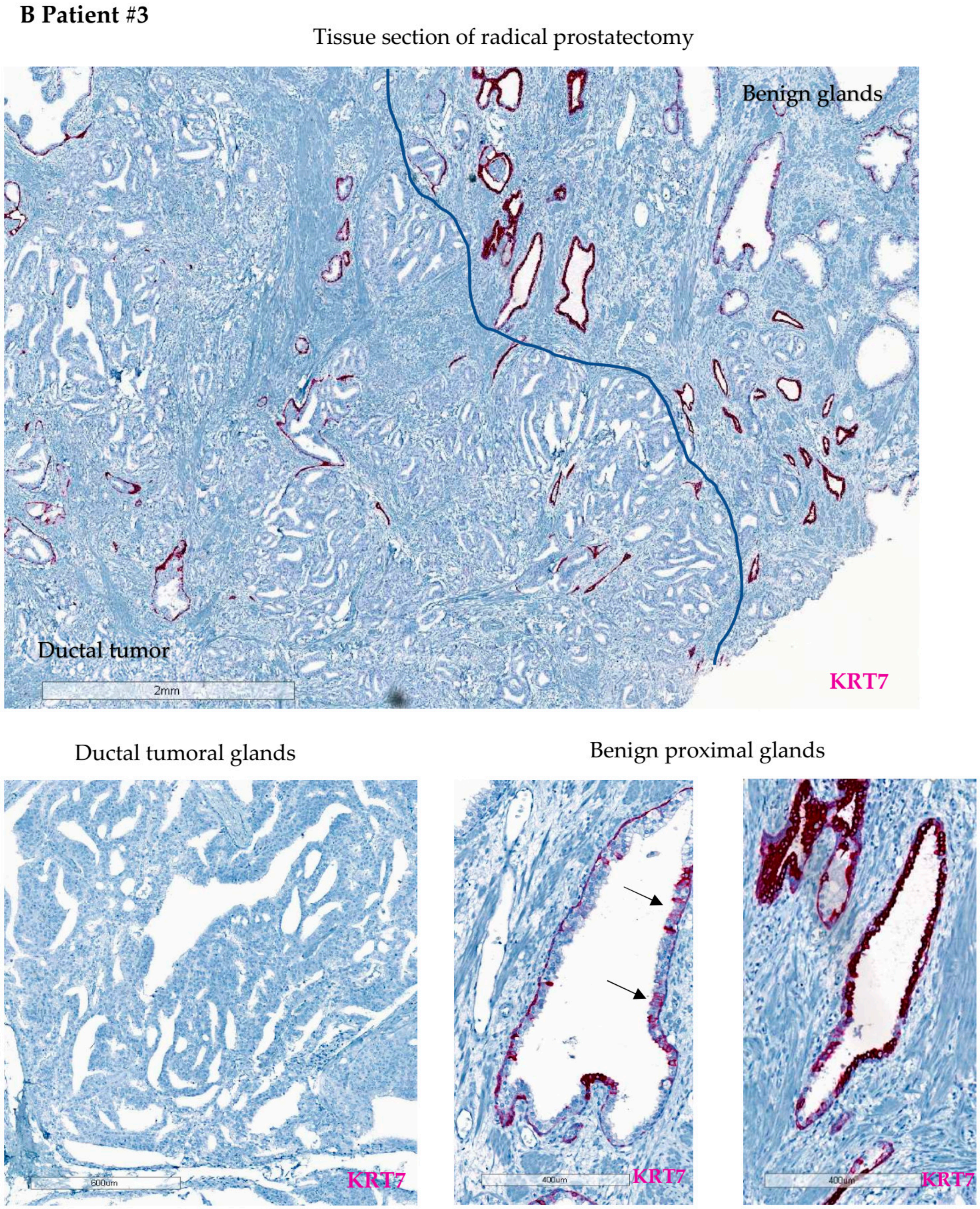

Figure S4. Additional examples of KRT7 staining by immunohistochemistry (IHC) using KRT7 rabbit SP52 clone on FFPE prostatic tissues section of radical prostatectomy from 2 other patients (Patient #2 and Patient #3) with prostatic ductal carcinoma. The blue line defines the separation between tumor and benign peri-tumoral glands. The KRT7 staining was elevated in peri-tumoral benign glands proximal to the tumor, but negative inside the tumor, and low in benign glands located distally. After magnification, positive KRT7 staining was confirmed mainly in the basal compartment

from benign proximal peri-tumoral glands, with a supra-basal staining identified with arrows, and negative in tumoral ductal glands. Positive staining was found in cells of the urethral urothelium, as a control. (**A**) Patient #2 (**B**) Patient #3.

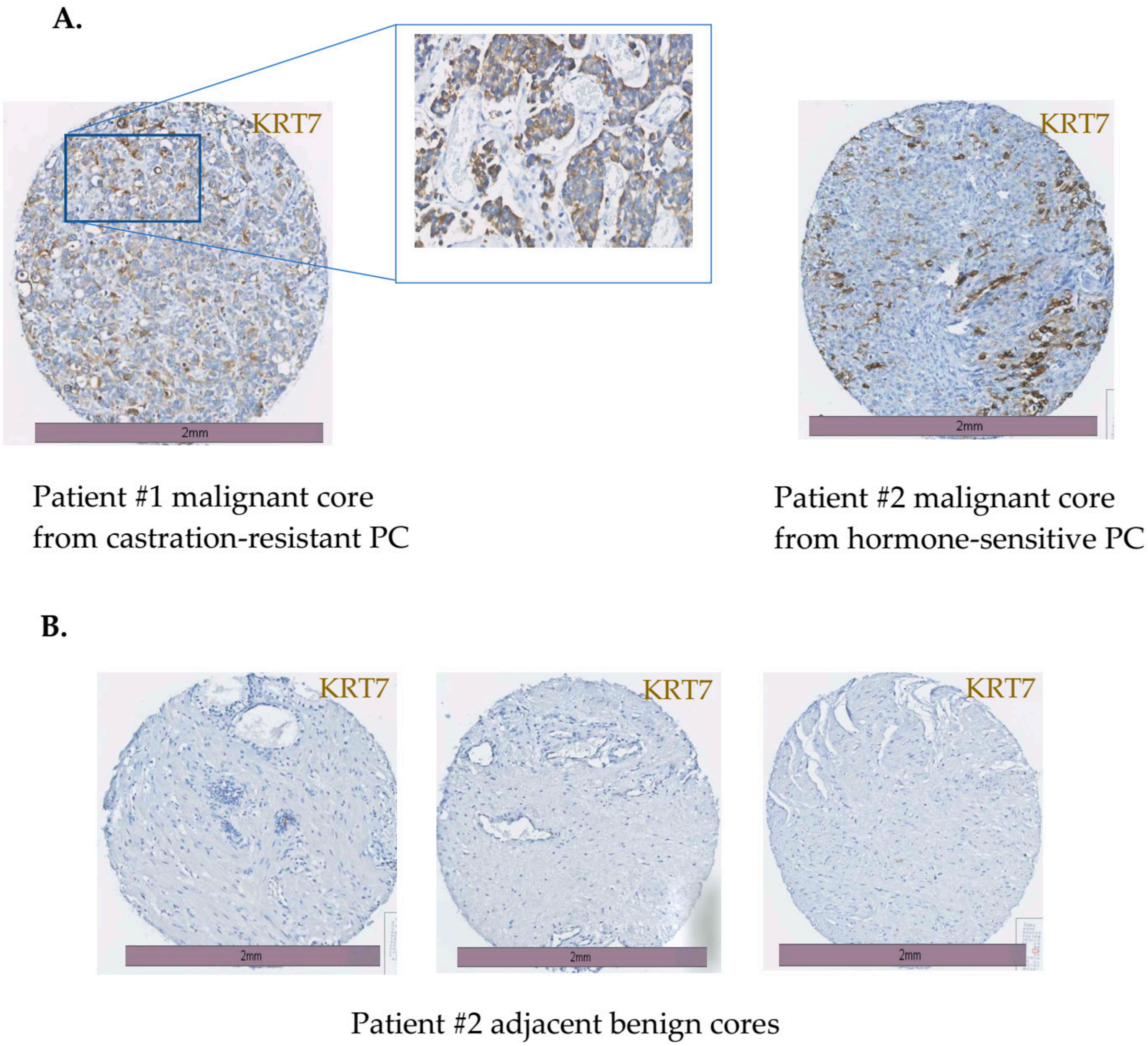

Figure S5. Detection of KRT7 expression by IHC in TURP cores. IHC assay with KRT7 staining using DAB kit detection on FFPE-cores from the TURP-TMA, n = 91 patients (clone OV-TL 12/30 mouse). (**A**) Malignant positive KRT7 cores from the 2 positive patients. KRT7 staining in luminal tumoral cells (patient # 1 with castration-resistant PC and patient # 2 with hormone-sensitive PC). (**B**) Corresponding adjacent benign cores from patient # 2. No evidence of KRT7 staining in basal cells from benign glands.

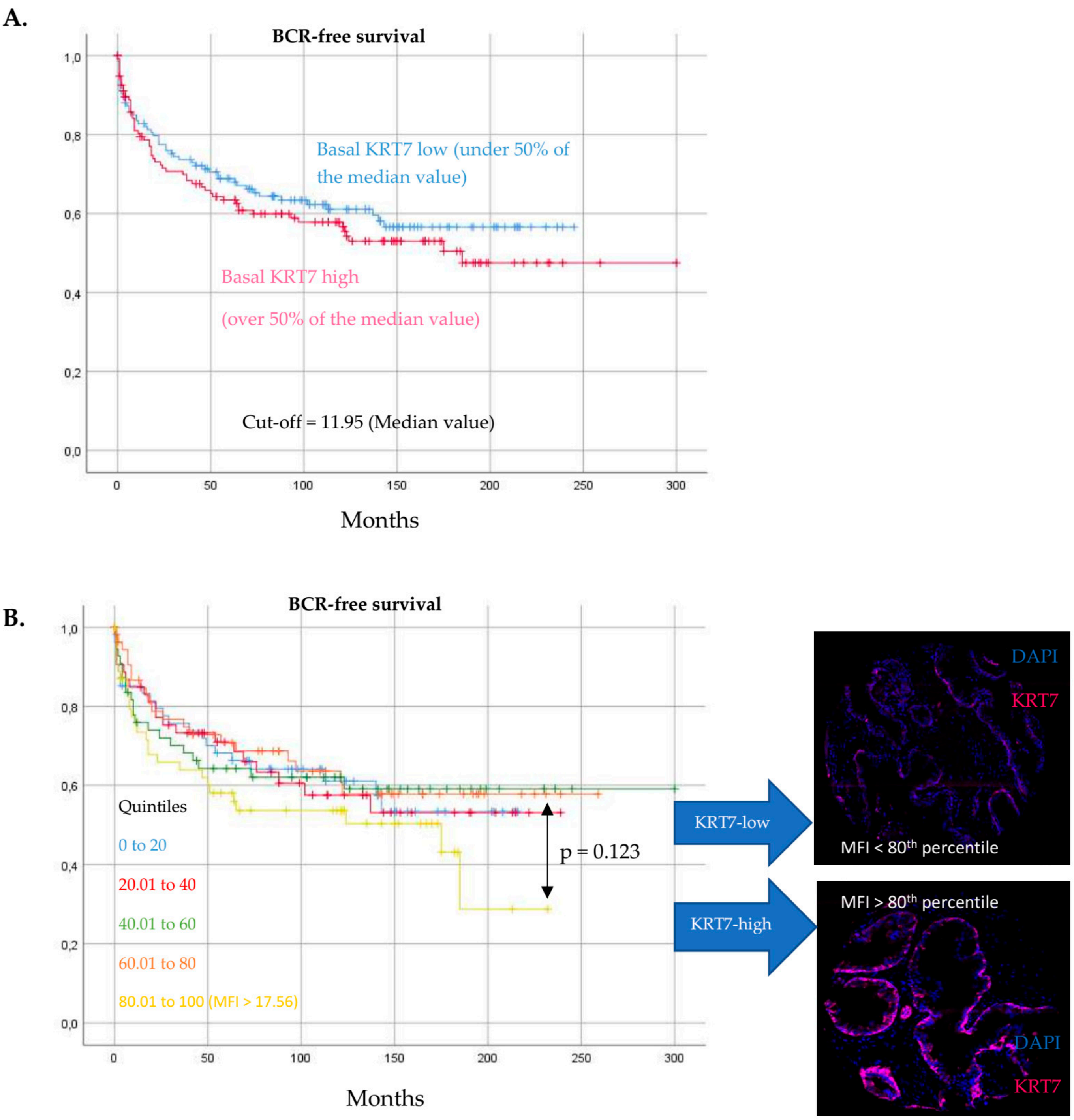

Figure S6. Impact of KRT7 basal expression on patient risk of biochemical recurrence (BCR) evaluated by Kaplan-Meier analysis coupled with a log-rank test, using various cut-offs of Mean Fluorescence Intensity (MFI). (**A**) Impact of high (over 50% of the median) and low (under 50% of the median) MFIs of KRT7 expression in basal epithelial cells from benign peri-tumoral glands on patient risk of BCR. (**B**) Potential of KRT7 expression in basal epithelial cells to predict BCR using a quintile method. Visualization of the selection of KRT7 MFI cut-off using a quintile method : trend is to a difference of BCR-free survival between KRT7-high patients (>80th percentile, group 4 in yellow) and the 4 other subgroups 0 to 3 (KRT7-low <80th percentile) (p = 0.123). A p-value > 0.05 was considered statistically not significant (NS).

Table S1. Clinical and pathological characteristics of the 285 patients who underwent radical prostatectomy.

Clinical and biological parameters	TF123 TMA cohort
Number of patients	285
Median age at RP, years (IQR)	63 (59–67)
Median PSA at diagnosis, ng/mL (IQR)	7 (5–10.8)
Pathological TNM	
2	201
3	75
4	9
Gleason score at RP	
GGG 1 (≤3 + 3)	140
GGG 2 (3 + 4)	93
GGG 3 (4 + 3)	19
GGG 4-5 (≥4 + 4)	29
Unknown	4
Positive margin (*n*, %)	95 (33.3)
Median follow-up, months (IQR)	129 (76–174)
Biochemical recurrence	
Number (*n*, %)	116 (40.7)
Median time to BCR, months (IQR)	11 (3–26)
Bone metastasis onset	
Number (*n*, %)	28 (9.8)
Median time to bone metastasis, months (IQR)	42 (20–83)
Overall survival	
Alive (*n*, %)	236 (82.8)
Death from other causes (*n*, %)	31 (10.9)
Death from PC (*n*, %)	18 (6.3)
Median time to PC specific mortality, months (IQR)	69 (49–150)

Abbreviations: BCR = biochemical recurrence; GGG = Gleason Grade Groups; IQR = interquartile range; TNM = Tumor Node Metastasis.
